# Network localization of genetic risk for schizophrenia and bipolar disorder

**DOI:** 10.1017/S0033291725101992

**Published:** 2025-10-03

**Authors:** Shanwen Yao, Fan Mo, Zhonghao Rao, Yu Shi, Jiajia Zhu, Yongqiang Yu

**Affiliations:** 1 Department of Radiology, The First Affiliated Hospital of Anhui Medical University, Hefei, China; 2 Research Center of Clinical Medical Imaging, Anhui Province, Hefei, China; 3 Anhui Provincial Institute of Translational Medicine, Hefei, China; 4 Anhui Provincial Key Laboratory for Brain Bank Construction and Resource Utilization, Hefei, China

**Keywords:** bipolar disorder, functional connectivity network mapping, genetic risk, neuroimaging, schizophrenia

## Abstract

**Background:**

There is a considerable overlap in clinical features and genetics between schizophrenia (SZ) and bipolar disorder (BD). Previous neuroimaging research has demonstrated common and distinct brain damage patterns between relatives (RELs) of SZ and BD patients, suggesting shared and differential genetic influences on the brain. Despite an increasing recognition that disorders localize better to distributed brain networks than individual brain regions, studies investigating network localization of genetic risk for SZ and BD are still lacking.

**Methods:**

To address this gap, we initially identified brain functional and structural damage locations in SZ- and BD-RELs from 103 published studies with 2364 SZ-RELs, 864 BD-RELs, and 4114 healthy controls. By applying novel functional connectivity network mapping to large-scale discovery and validation resting-state functional MRI datasets, we mapped these affected brain locations to four disorder-susceptibility networks.

**Results:**

SZ-susceptibility functional damage network primarily involved the executive control and salience networks, while its BD-counterpart principally implicated the default mode and basal ganglia networks. SZ-susceptibility structural damage network predominantly involved the auditory and default mode networks, yet its BD-counterpart mainly implicated the language and executive control networks. Although these networks showed cross-disorder inconsistencies when focusing on either imaging modality alone, the combined SZ- and BD-susceptibility brain damage networks had a substantially increased spatial similarity.

**Conclusions:**

These findings may support the concept that SZ and BD represent distinct diagnostic categories from a neurobiological perspective, helping to clarify the common network substrates via which the shared genetic mechanisms underlying both disorders give rise to their overlapping clinical phenotypes.

## Introduction

Schizophrenia (SZ) and bipolar disorder (BD) have been conceptualized as distinct chronic psychiatric disorders in the current diagnostic system and are often studied individually (Barch, 2020). However, it is now clear that the two conditions share some common clinical features and genetic risk factors (Grande et al., 2016; Kahn et al., 2015; Murray et al., 2004; Whalley, 2023), potentially challenging the traditional diagnostic categories in psychiatry. For instance, while psychotic symptoms are central to SZ and affective symptoms are thought to be more characteristic of BD, both types of clinical symptoms can occur in the context of either diagnosis (Bora, 2016; Kempf et al., 2005; Murray et al., 2004). In parallel, genetics evidence from genome-wide and transcriptome-wide association studies has shown that several genes are implicated in the risk for both SZ and BD (Cross-Disorder Group of the Psychiatric Genomics Consortium, 2013; Gandal et al., 2018; Lichtenstein et al., 2009; Prata et al., 2019; Schizophrenia Working Group of the Psychiatric Genomics Consortium, 2014; Stahl et al., 2019), emphasizing a shared genetic basis. Despite this growing literature, whether SZ and BD are the clinical outcomes of discrete or common causative processes is still highly debated. Critically, the intermediate phenotype concept represents a strategy for characterizing neural abnormalities in psychiatric disorders, which may help to bridge the gap between genetic susceptibility and clinical phenotype (Meyer-Lindenberg & Weinberger, 2006; Rasetti & Weinberger, 2011).

Close relatives (RELs) of SZ or BD patients share up to 50% of the genes of the patients and thus are at greater genetic risk for developing these disorders (Maier et al., 2006). The study of unaffected RELs is not influenced by many confounding factors pertinent to the disease, such as exposure to acute illness status, medication use, illness chronicity, and substance abuse. Therefore, investigations into brain functional and structural alterations in unaffected RELs of SZ or BD patients may help to elucidate the neurobiological mechanisms underlying genetic vulnerability to these diseases. Neuroimaging, and magnetic resonance imaging (MRI) in particular, has strengthened its position as the most widely applied tool in psychiatry research (Li et al., 2021; Luo et al., 2022). Although appropriately scrutinized for irreproducible results, brain functional and structural measures derived from neuroimaging techniques are closer to genetic effects relative to the diagnosis itself and have revealed important insights into the neuropathology of psychiatric disorders (Etkin, 2019; Lui et al., 2016). By leveraging neuroimaging measures, extensive research has established the presence of brain functional and structural damage in either SZ- or BD-RELs (Cao et al., 2016; Cattarinussi et al., 2019; Cattarinussi et al., 2022; de Zwarte et al., 2019; Ivleva et al., 2013; McDonald et al., 2006; Rasetti et al., 2011; Saarinen et al., 2020; Scognamiglio & Houenou, 2014; Skudlarski et al., 2013; Zhang et al., 2020). Moreover, a recent coordinate-based neuroimaging meta-analysis has demonstrated common and distinct brain damage patterns between SZ- and BD-RELs, suggesting shared and differential genetic influences on the brain (Cattarinussi et al., 2022).

The conventional coordinate-based meta-analyses of neuroimaging data evaluate the spatial convergence of anatomical regions associated with a given disorder across different studies (Eickhoff et al., 2009). However, there is an increasing agreement that many disorders and symptoms map to connected brain networks better than they do to single brain regions (Fornito et al., 2015; Fox, 2018; Taylor et al., 2021), as neuropathological phenomena do not act in isolation, but are instead interconnected via distributed networks. Functional connectivity network mapping (FCNM) is a novel and well-validated approach that can localize a disease, symptom, or psychological process to a common brain network, by integrating brain locations of interest (e.g. lesion, structural damage, functional abnormality, and neural activation) with the human brain connectome derived from large-scale functional neuroimaging data (Cheng et al., 2025; Darby et al., 2019; Joutsa et al., 2022; Mo et al., 2024; Peng et al., 2022; Taylor et al., 2023; Xu et al., 2024 **;** Zhang et al., 2024). This network-based framework has been broadly applied to neuropsychiatric conditions and has enjoyed significant success in advancing our understanding of disease mechanisms from a network perspective (Cotovio et al., 2020; Cotovio et al., 2022; Darby et al., 2019; Jones et al., 2023; Padmanabhan et al., 2019; Taylor et al., 2021; Taylor et al., 2023; Tetreault et al., 2020; Trapp et al., 2023; Zhukovsky et al., 2021). Despite this progress, studies investigating network localization of genetic risk for SZ and BD are still lacking.

**Figure 1.**
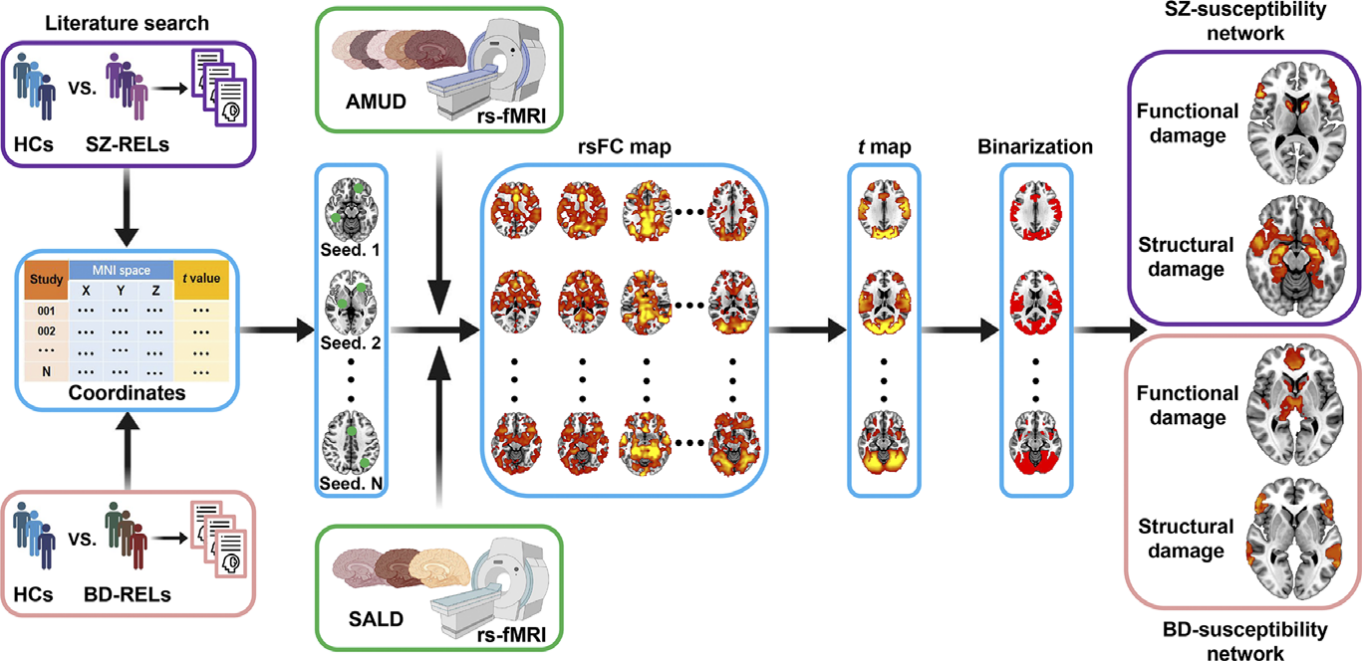
Study design and analytical procedure. We initially synthesized published literature to identify brain functional and structural damage locations in SZ- and BD-RELs. By combining these affected brain locations with large-scale discovery (AMUD) and validation (SALD) rs-fMRI datasets, we then adopted the FCNM approach to construct disorder-susceptibility networks (i.e. SZ- and BD-susceptibility functional and structural damage networks). Specifically, spheres centered at each coordinate of a contrast were first created and merged together to generate a contrast-specific combined seed mask. Second, based on the rs-fMRI data, we computed a contrast seed-to-whole brain rsFC map for each subject. Third, the subject-level rsFC maps were entered into a voxel-wise one-sample *t* test to identify brain regions functionally connected to each contrast seed. Fourth, the resulting group-level *t* maps were thresholded and binarized at *p* < 0.05 corrected for multiple comparisons using a voxel-level FDR method. Finally, the binarized maps of the contrasts were overlaid to produce four network probability maps, which were thresholded at 60% to yield SZ-susceptibility functional and structural as well as BD-susceptibility functional and structural damage network, respectively. Abbreviations: AMUD, Anhui Medical University Dataset; BD, bipolar disorder; FCNM, functional connectivity network mapping; FDR, false discovery rate; HCs, healthy controls; RELs, relatives; rs-fMRI, resting state functional magnetic resonance imaging; rsFC, resting state functional connectivity; SALD, Southwest University Adult Lifespan Dataset; SZ, schizophrenia.

To address this missing gap, we adopted the FCNM approach to investigate the brain network substrates underlying SZ- and BD-RELs, potentially unifying the heterogeneous findings across prior neuroimaging studies from a network perspective. Specifically, we initially synthesized published literature to identify brain functional and structural damage locations in SZ- and BD-RELs. By combining these affected brain locations with large-scale discovery and validation resting-state functional magnetic resonance imaging (fMRI) datasets, we then adopted the FCNM approach to construct four disorder-susceptibility networks (i.e. SZ- and BD-susceptibility functional and structural damage networks). In addition, we assessed the spatial similarity between the SZ- and BD-susceptibility networks to examine shared and differential genetic effects. Schematic representation of the study design and analytical procedure is provided in Figure 1. Building on previous evidence, we hypothesized that differences and commonalities would exist in the susceptibility networks across disorders and imaging modalities.

## Methods

### Study selection and classification

We performed a comprehensive and systematic literature search in PubMed and Web of Science to identify relevant studies examining brain functional or structural damage in SZ- or BD-RELs, published before February 1, 2023, following the Meta-analysis of Observational Studies in Epidemiology guidelines (Stroup et al., 2000). The study selection process is detailed in Supplementary Methods and Figure S1 in the Supplementary Materials. The protocol was registered on PROSPERO (https://www.crd.york.ac.uk/PROSPERO/, registration number: CRD42023467611). A total of 103 studies with 2364 SZ-RELs, 864 BD-RELs, and 4114 healthy controls (HCs) were selected and included in our analysis. The number of studies for each imaging modality was (SZ-RELs/BD-RELs): 81 task fMRI studies (*n* = 58/23) and 26 voxel-based morphometry (VBM) studies (*n* = 16/10). Sample information of the selected studies is provided in Tables S1–S5 in the Supplementary Materials. Since a single study may contain multiple contrasts (i.e. brain activation or volume differences between SZ-RELs and HCs or between BD-RELs and HCs), we focused our analysis on contrasts rather than studies. Coordinates of peak voxels of significant clusters reported in each contrast were extracted, and coordinates in Talairach space were converted to Montreal Neurological Institute (MNI) space.

### Discovery and validation datasets

Our study used Anhui Medical University Dataset (AMUD) as a discovery dataset and Southwest University Adult Lifespan Dataset (SALD) (Wei et al., 2018) as a cross-scanner validation dataset. AMUD included 656 healthy adults of Chinese Han and right handedness (396 female, mean 26.57 ± 8.57 years), who were enrolled from local universities and communities through poster advertisements. Participants with neuropsychiatric or severe somatic disorders, a history of head injury with consciousness loss, MRI contraindications, or a family history of psychiatric diseases among first-degree relatives were excluded. This study was approved by the ethics committee of The First Affiliated Hospital of Anhui Medical University, and all participants provided written informed consent after being given a complete description of the study. SALD included 329 healthy adults (207 female, mean 37.81 ± 13.79 years). For the SALD dataset, the exclusion criteria included MRI contraindications, current psychiatric or neurological disorders, use of psychiatric drugs within 3 months, pregnancy, or a history of head trauma. Full details regarding the sample have been described in the data descriptor publication (Wei et al., 2018). It is noteworthy that all included participants were restricted to an age range of 18–60 years to exclude the potential effects of neurodevelopment and neurodegeneration. Demographic information of the discovery and validation datasets is provided in Table S6 in the Supplementary Materials.

The authors assert that all procedures contributing to this work comply with the ethical standards of the relevant national and institutional committees on human experimentation and with the Helsinki Declaration of 1975, as revised in 2008. All procedures involving human subjects in the AMUD dataset were conducted following approval by the ethics committee of The First Affiliated Hospital of Anhui Medical University (approval number 20200094), and all participants provided written informed consent after being given a complete description of the study. As for the SALD dataset, it is a publicly available resource, and detailed ethical information can be found at http://fcon_1000.projects.nitrc.org/indi/retro/sald.html.

### fMRI data acquisition and preprocessing

Resting state fMRI data of AMUD were collected on a 3.0-Tesla General Electric Discovery MR750w scanner, and those of SALD on a 3.0-Tesla Siemens Trio scanner. The fMRI parameters of the two datasets are provided in Table S7 in the Supplementary Materials. Participants with poor image quality (e.g. visible artifacts) and incomplete brain coverage were excluded.

Resting-state fMRI data were preprocessed using Statistical Parametric Mapping software (SPM12, http://www.fil.ion.ucl.ac.uk/spm) and Data Processing & Analysis for Brain Imaging (DPABI, http://rfmri.org/dpabi) (Yan et al., 2016). The first 10 volumes for each participant were discarded to allow the signal to reach equilibrium and the participants to adapt to the scanning noise. The remaining volumes were corrected for the acquisition time delay between slices. Then, realignment was performed to correct the motion between time points. Head motion parameters were computed by estimating the translation in each direction and the angular rotation on each axis for each volume. All participants’ BOLD data were within the defined motion thresholds (i.e. maximal translational or rotational motion parameters less than 2 mm or 2^°^). We also calculated frame-wise displacement (FD), which indexes the volume-to-volume changes in head position. Several nuisance covariates (the linear drift, the estimated motion parameters based on the Friston-24 model, the spike volumes with FD >0.5 mm, the global signal, the white matter signal, and the cerebrospinal fluid signal) were regressed out from the data. Since global signal regression can enhance the detection of system-specific correlations and improve the correspondence to anatomical connectivity (Murphy & Fox, 2017), we included this step in the preprocessing of resting state fMRI data. Next, the datasets were band-pass filtered using a frequency range of 0.01–0.1 Hz. In the normalization step, individual structural images were first coregistered with the mean functional images; the transformed structural images were then segmented and normalized to MNI space using a high-level nonlinear warping algorithm, that is, the diffeomorphic anatomical registration through exponentiated Lie algebra technique (Ashburner, 2007). Then, each filtered functional volume was spatially normalized to MNI space using the deformation parameters estimated during the earlier step and resampled into a 3-mm isotropic voxel. Finally, all data were spatially smoothed with a Gaussian kernel of 6 × 6 × 6 mm^3^ full width at half maximum.

### Functional connectivity network mapping

The FCNM approach was employed to construct four disorder-susceptibility networks (i.e. SZ- and BD-susceptibility functional and structural damage networks) based on the extracted coordinates of brain functional and structural damage in SZ- and BD-RELs, respectively (Figure 1). First, 4-mm radius spheres centered at each coordinate of a contrast were created and merged together to generate a combined seed mask specific to that contrast (henceforth referred to as the contrast seed). Second, based on the preprocessed resting state fMRI data of AMUD, we computed a contrast seed-to-whole brain functional connectivity (FC) map for each subject, by calculating Pearson’s correlation coefficients between time courses of the contrast seed and each voxel within the whole brain, followed by Fisher’s *Z* transformation to improve normality. Third, the 656 subject-level FC maps were entered into a voxel-wise one-sample *t* test to identify brain regions functionally connected to each contrast seed. Note that we only considered positive FC as the biological meaning of negative FC is still a matter of debate (Murphy et al., 2009; Murphy & Fox, 2017). Fourth, the resulting group-level *t* maps were thresholded and binarized at *p* < 0.05 corrected for multiple comparisons using a voxel-level false discovery rate (FDR) method. Finally, the binarized maps of the contrasts were overlaid to produce four network probability maps, which were thresholded at 60% to yield SZ- and BD-susceptibility functional and structural damage networks, respectively. In the interest of completeness, we combined the resultant disorder-susceptibility functional and structural damage networks to further obtain disorder-susceptibility brain damage networks.

To quantify the similarity of network patterns between disorders, we assessed the spatial overlap between the SZ- and BD-susceptibility networks by calculating a Dice coefficient, defined as 2 × (overlapping voxels)/(network #1 voxels) + (network #2 voxels). A higher Dice coefficient indicates more similar networks.

### Relation to canonical brain networks

For ease of interpretability, we examined the spatial relationships between the disorder-susceptibility networks and 14 well-established canonical brain networks (Shirer et al., 2012). The proportion of overlapping voxels between each disorder-susceptibility network and a canonical network to all voxels within the corresponding canonical network was calculated to quantify their spatial relationship.

### Validation analyses

We conducted several validation analyses to test the robustness of our results. First, to exclude the impact of dataset selection, we carried out the same analyses based on an independent validation dataset (i.e. cross-scanner SALD). Second, to determine whether our findings were influenced by seed size, we repeated the FCNM procedure using 1 mm and 7 mm radius spheres. Finally, to further exclude the influence of neurodegeneration, we repeated our analyses in the young adults within an age range of 18–30 years.

## Results

### SZ-susceptibility functional damage network

**Figure 2.**
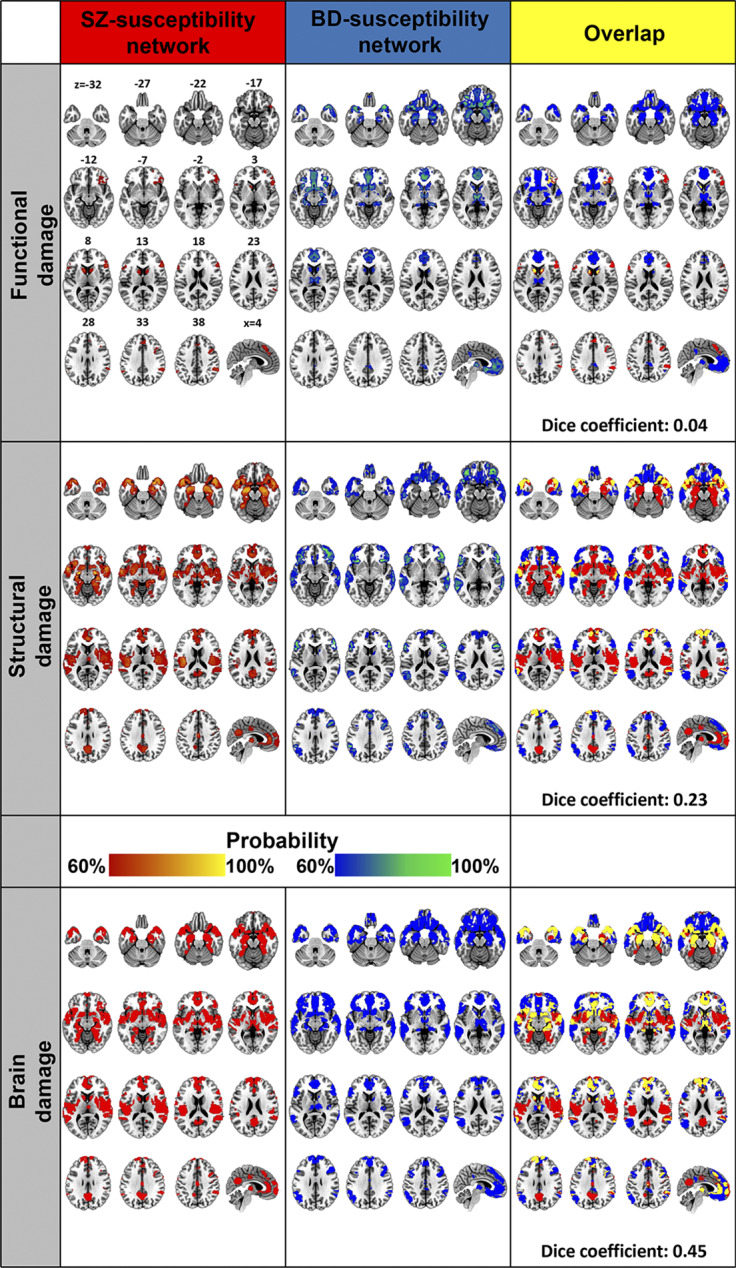
Schizophrenia and bipolar disorder susceptibility networks. Left panel: SZ-susceptibility functional, structural, and combined brain damage networks. Middle panel: BD-susceptibility functional, structural, and combined brain damage networks. Right panel: spatial overlap between SZ- and BD-susceptibility networks. Abbreviations: BD, bipolar disorder; SZ, schizophrenia.

**Figure 3.**
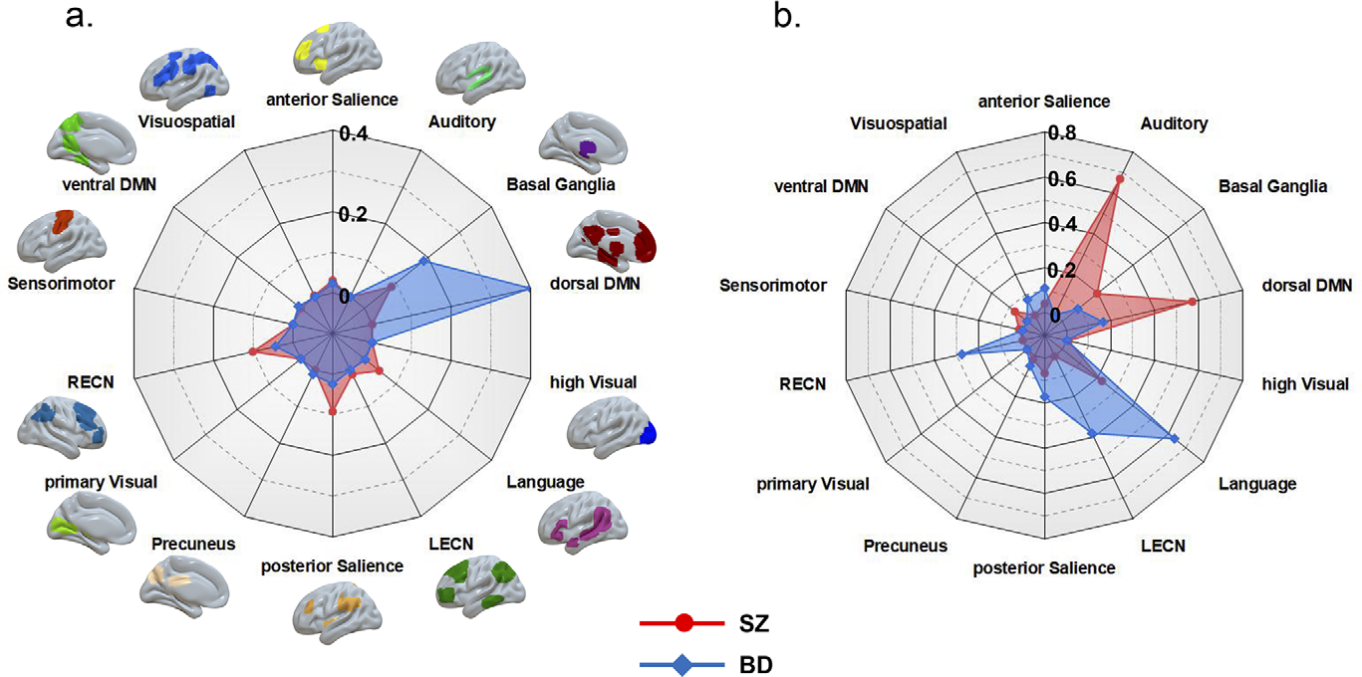
Schizophrenia and bipolar disorder susceptibility networks in relation to canonical brain networks. (a) Functional damage networks. (b) Structural damage networks. Polar plots illustrate the proportion of overlapping voxels between each disorder-susceptibility network and a canonical network to all voxels within the corresponding canonical network. Abbreviations: BD, bipolar disorder; DMN, default mode network; LECN, left executive control network; RECN, right executive control network; SZ, schizophrenia.

SZ-susceptibility functional damage network comprised a sparsely distributed set of brain regions including the bilateral dorsal medial prefrontal cortex, inferior parietal lobule, inferior frontal gyrus and caudate, and the right superior frontal gyrus, temporal pole, orbitofrontal cortex and insula (Figure 2). With regard to canonical brain networks, SZ-susceptibility functional damage network primarily involved the right executive control (overlapping proportion: 10.2%) and posterior salience (9.2%) networks (Figure 3a).

### BD-susceptibility functional damage network

BD-susceptibility functional damage network mainly consisted of the bilateral ventral medial prefrontal cortex, anterior and middle cingulate cortex, temporal pole, medial temporal cortex, insula, thalamus, hippocampus, amygdala, and striatum (Figure 2). As to canonical networks, BD-susceptibility functional damage network principally implicated the dorsal default mode (41.5%) and basal ganglia (18.7%) networks (Figure 3a).

### SZ-susceptibility structural damage network

SZ-susceptibility structural damage network comprised widespread brain areas chiefly including the bilateral anterior, middle, and posterior cingulate cortex, ventral medial prefrontal cortex, temporal pole, superior temporal gyrus, insula and operculum, medial temporal cortex, thalamus, hippocampus, amygdala, and striatum (Figure 2). With respect to canonical networks, SZ-susceptibility structural damage network predominantly involved the auditory (66.9%) and dorsal default mode (57.1%) networks (Figure 3b).

### BD-susceptibility structural damage network

BD-susceptibility structural damage network was primarily composed of the bilateral dorsal medial prefrontal cortex, orbitofrontal cortex, lateral prefrontal cortex, lateral temporal cortex, and posterior parietal cortex, and the right caudate (Figure 2). Regarding canonical networks, BD-susceptibility structural damage network mainly implicated the language (63.5%) and left executive control (38.2%) networks (Figure 3b).

### Network similarity between disorders

When focusing on a single imaging modality, we observed low spatial similarities between SZ- and BD-susceptibility functional damage networks (Dice coefficient = 0.04) and between SZ- and BD-susceptibility structural damage networks (Dice coefficient = 0.23) (Figure 2). By combining the functional and structural damage networks, we found that the resultant SZ- and BD-susceptibility brain damage networks had a substantially increased spatial similarity (Dice coefficient = 0.45), (Figure 2). The overlapping regions included the medial prefrontal cortex, anterior cingulate cortex, temporal pole, hippocampus, amygdala, thalamus, caudate, and nucleus accumbens. The SZ-specific regions included superior temporal gyrus, insula and operculum, medial temporal cortex, posterior cingulate cortex, putamen, and globus pallidus. The BD-specific regions included the orbitofrontal cortex, lateral temporal cortex, lateral prefrontal cortex, and posterior parietal cortex.

### Validation analyses

First, the SZ- and BD-susceptibility networks derived from the validation SALD dataset were similar to those from the discovery AMUD dataset, with subtle differences largely attributed to variation in sample sizes (656 vs. 329) (Figure S2 in the Supplementary Materials). Second, when repeating the FCNM procedure using 1 mm and 7 mm radius spheres, we observed that the resulting SZ- and BD-susceptibility networks were nearly identical to those using the 4 mm radius sphere (Figures S3 and S4 in the Supplementary Materials). Finally, our analyses in the young adults within an age range of 18–30 years yielded results similar to those of the main analyses in all participants (Figure S5 in the Supplementary Materials). These findings verified the robustness of our results to distinct datasets and methodological differences.

## Discussion

By a combination of the novel FCNM approach and large-scale human brain connectome data, this study investigated network localization of genetic risk for SZ and BD based on brain damage locations in SZ- and BD-RELs reported in previous literature. We found four disorder-susceptibility networks with respect to different imaging modalities. SZ-susceptibility functional damage network primarily involved the executive control and salience networks, while its BD-counterpart principally implicated the default mode and basal ganglia networks. SZ-susceptibility structural damage network predominantly involved the auditory and default mode networks, yet its BD counterpart mainly implicated the language and executive control networks. These disparities endorse the concept that SZ and BD represent distinct diagnostic entities from the perspective of network localization. Although neither SZ-susceptibility functional nor structural damage networks were similar to their BD-counterparts, the combined SZ- and BD-susceptibility brain damage networks had quite similar spatial distributions, which may be the expression of the shared genetic mechanism underlying both disorders and could contribute to their overlapping clinical features.

Neuroimaging has been appropriately scrutinized for irreproducible results (Poldrack et al., 2017), which has limited its clinical application in psychiatric diagnosis and treatment (Etkin, 2019). This lack of reproducibility can be ascribed to a number of factors, such as small samples, clinical heterogeneity, and methodological differences (Etkin, 2019; Poldrack et al., 2017). Conventional coordinate-based neuroimaging meta-analyses offer a useful method to examine the spatial convergence of anatomical regions related to a given disorder across multiple studies (Eickhoff et al., 2009). Nevertheless, it is generally accepted that neuropathological phenomena or neural processes do not act in isolation, but are instead interconnected via distributed brain networks (Fornito et al., 2015), such that localization of a disorder, symptom, or psychological process has recently shifted from a predominant regional approach to an updated connectomic paradigm. The conceptual and methodological advancements largely benefit from the development of the FCNM approach that integrates brain locations of interest (e.g. lesion, structural damage, functional abnormality, and neural activation) with large-scale human brain connectome data (Darby et al., 2019; Joutsa et al., 2022; Peng et al., 2022; Taylor et al., 2023). By use of FCNM, researchers have mapped various disorders, many of which have eluded conventional regional localization, to specific brain networks (Cotovio et al., 2020; Cotovio et al., 2022; Darby et al., 2019; Jones et al., 2023; Padmanabhan et al., 2019; Taylor et al., 2021; Taylor et al., 2023; Tetreault et al., 2020; Trapp et al., 2023; Zhukovsky et al., 2021), with the assumption that abnormalities in multiple different brain locations that cause the same disorder can localize to a common network. With respect to neural correlates of genetic risk for SZ and BD, a recently published coordinate-based neuroimaging meta-analysis found reduced thalamic volume in both SZ- and BD-RELs, corticostriatal-thalamic changes in SZ-RELs, and thalamocortical and limbic alterations in BD-RELs, indicating shared and differential genetic influences on regional brain function and structure (Cattarinussi et al., 2022). Complementing and extending this prior study, we used the FCNM approach to disentangle the nature and extent of SZ and BD genetic effects on the brain from a network perspective. Our work, in conjunction with these previous network-based efforts, highlights the potential usefulness of FCNM as a promising tool to improve our understanding of disease mechanisms.

SZ-susceptibility functional damage network primarily involved the executive control and salience networks. The executive control network is typically responsible for processes related to goal-directed behaviors, working memory, and attention control (Chen et al., 2013; Menon, 2011; Reuveni et al., 2023; Shen et al., 2020). The disruption of the executive control network has been well documented in SZ (Anhøj et al., 2018; Kraguljac et al., 2016; Supekar et al., 2019). The salience network is crucial for sustaining human emotion and cognition, especially during the detection and processing of salient information (Cai et al., 2021; Craig, 2002; Seeley et al., 2007). Salience network deficits, occurring across different stages of SZ (Huang et al., 2020; Pu et al., 2012; Wang et al., 2016), have been shown to associate with SZ core symptoms, such as hallucinations and delusions (Palaniyappan et al., 2011; Palaniyappan & Liddle, 2012; Pu et al., 2012; White et al., 2010). Furthermore, accumulating evidence suggests the critical role of the salience network in switching between the executive control and default mode networks (Molnar-Szakacs & Uddin, 2022; Sridharan et al., 2008), highlighting that the functional organization and dynamic interaction of these three networks underlie a wide range of mental disorders including SZ, resulting in the triple network model of psychopathology (Hogeveen et al., 2018; Menon, 2011, 2018; Menon et al., 2023). Different from SZ, BD-susceptibility functional damage network principally implicated the default mode and basal ganglia networks. The default mode network, whose activity is maximum at rest and suppressed during tasks, reflects intrinsic or endogenous neural activity (Gusnard et al., 2001; Mason et al., 2007). Moreover, the high heritability of default mode network function makes it a candidate intermediate phenotype in the study of the genetic basis of psychiatric illnesses (Glahn et al., 2010; Korgaonkar et al., 2014; Xu et al., 2017). Indeed, default mode network abnormalities have been evident in BD patients and their RELs (Doucet et al., 2017; Meda et al., 2012; Meda et al., 2014; Ongür et al., 2010). Despite the traditional view of its prominent involvement in motor learning and movement execution, knowledge about basal ganglia physiology has evolved during the last decades and this network is now considered as a key regulator of important cognitive and emotional processes (Mancini et al., 2021). The frequently reported neural circuits (e.g. the corticolimbic and prefrontal–striatal–thalamic circuits) affected in BD commonly involve the basal ganglia (Brooks & Vizueta, 2014; Strakowski et al., 2005; Vai et al., 2014; Vai et al., 2019; Zhang et al., 2022), emphasizing its significant contribution to the pathophysiology of BD.

SZ-susceptibility structural damage network predominantly involved the auditory and default mode networks. The auditory network is engaged in auditory perception and processing. Extensive research has revealed structural and functional abnormalities in the auditory network in SZ patients and their RELs (Cui et al., 2017; Joo et al., 2020; Liemburg et al., 2012; Oertel-Knöchel et al., 2014; Rajarethinam et al., 2004). Numerous studies have also demonstrated an intimate link between auditory network deficits and auditory hallucinations (Ćurčić-Blake et al., 2017; Mallikarjun et al., 2018; Oertel-Knöchel et al., 2014; Richards et al., 2021; Upthegrove et al., 2016; Xie et al., 2023), a core psychotic symptom affecting up to 60–80% of SZ patients. Regarding the default mode network, its relationships with SZ as well as psychotic symptoms have been well established (Bluhm et al., 2007; Camchong et al., 2011; Garrity et al., 2007; Lynall et al., 2010; Rotarska-Jagiela et al., 2010; Zhou et al., 2007). van Buuren et al. (2012) also found that healthy siblings of SZ patients exhibited abnormal intrinsic connectivity within the default mode network. Distinct from SZ, BD-susceptibility structural damage network mainly implicated the language and executive control networks. The language network predominantly implicates Broca’s and Wernicke’s areas in the inferior frontal gyrus and temporal–parietal junction of the left hemisphere, respectively. There is strong evidence for structural and functional alterations in these brain regions in BD patients and their RELs (Drobinin et al., 2019; Hafeman et al., 2014; Hajek et al., 2013; Liang et al., 2022; Romeo et al., 2022; Stoddard et al., 2016). Although BD is characterized by emotion processing abnormalities, BD patients and their RELs present with prominent executive function impairment and its underlying neural correlate, that is, executive control network dysfunction (Arts et al., 2008; Singh et al., 2014; Wu et al., 2013).

When combining functional and structural damage networks, we found that the resultant SZ- and BD-susceptibility brain damage networks showed substantially increased spatial similarity, with the convergent abnormalities primarily localized to the medial prefrontal cortex, anterior cingulate cortex, temporal pole, hippocampus, amygdala, thalamus, caudate, and nucleus accumbens. These affected regions across disorders are consistent with findings from previous transdiagnostic studies, emphasizing the involvement of the salience and subcortical networks (Caseras et al., 2015; Goodkind et al., 2015; McIntosh et al., 2004; Rimol et al., 2010). The Psychiatric Genomics Consortium and other large-scale genome-wide association studies (GWAS) have identified multiple genetic variants associated with the risk for SZ and BD (Schizophrenia Working Group of the Psychiatric Genomics Consortium, 2014; Stahl et al., 2019). Moreover, numerous imaging genetics studies have combined genetic data with brain MRI to investigate how these risk genes contribute to brain functional and structural abnormalities. For example, large-scale imaging genetics research has found that several psychiatric risk variants significantly influence subcortical brain structure, suggesting that genetic risk may confer disease susceptibility through its impact on the structural architecture of specific brain regions (Hibar et al., 2015). Additionally, imaging genetics studies in SZ reveal that carriers of multiple risk genes and genetic variants, including CACNA1C, NRG1, DRD2 (rs1076560), COMT (Val158Met), miR-137, and others, as well as high-risk populations, exhibit significant brain functional and structural abnormalities in regions such as the prefrontal cortex, temporal cortices, precuneus, thalamus, and striatum (Erk et al., 2014; Grimm et al., 2014; Jagannathan et al., 2010; Jamadar et al., 2011; Liu et al., 2020; Luykx et al., 2017; Sambataro et al., 2013; Tunbridge et al., 2013; van Erp et al., 2014; Wright et al., 2016). Although imaging genetics studies in BD are relatively limited in sample size, existing research indicates that BD-related risk genes, such as CACNA1C and ANK3, are associated with functional and structural abnormalities in prefrontal and limbic regions (Dima et al., 2013; Erk et al., 2014; Perrier et al., 2011; Soeiro-de-Souza et al., 2017). The SZ- and BD-susceptibility brain damage networks identified in our work show high spatial concordance with these imaging genetics findings.

Notably, we observed pronounced dissociations between the functional and structural findings in both disorders. While the relationship between brain functional and structural abnormalities in psychiatric disorders remains incompletely understood, emerging evidence suggests both compensatory network reorganization and independent pathological processes. The breakdown of functional connectivity between brain regions can drive adaptive changes, such as over- or under-utilization of established pathways without requiring physical fiber disruption (Skudlarski et al., 2010), whereas structural and functional abnormalities may also arise through distinct mechanisms, as evidenced by their dissociations in SZ and depression (Gong et al., 2016; Guo et al., 2015, 2014; Ren et al., 2013; Zhuo et al., 2017). Our findings demonstrate that the dissociations between functional and structural brain damage networks are already present in unaffected relatives of SZ and BD patients, indicating that modality-specific vulnerability patterns independently shape disease neuropathology.

There are several limitations to our study. First, we utilized resting state fMRI data from healthy adults to examine network localization of genetic risk for SZ and BD. It seems preferable to use fMRI data from samples well matching the SZ- and BD-RELs in terms of demographic and clinical features. Nevertheless, earlier studies have demonstrated that sample selection makes little impact on network localization (Boes et al., 2015; Fox et al., 2014; Horn et al., 2017). Second, our study design was retrospective rather than prospective. Some RELs included in our analyses, adolescents in particular, may ultimately develop a major psychiatric disorder, which may influence our interpretation. Future prospective investigation is warranted to verify our preliminary findings. Third, a less conservative overlapping threshold (60%) was adopted to identify brain regions that were functionally connected to 60% of the contrast seeds since many sources of variance, for example, differences of the selected studies in their statistical power, participants’ age ranges, and scanners, may prevent us from finding a common brain network. There has been no consensus yet on how to account for these factors. Further investigation of their influences, in concert with analytical advances in the future, will help address this issue. Fourth, our analysis was restricted to task-based fMRI, while emerging evidence suggests resting-state and dynamic functional connectivity may provide complementary mechanistic insights into neuropsychiatric disorders (Wang et al., 2024; You et al., 2022). Future studies should incorporate these modalities to fully characterize brain network alterations in RELs of SZ and BD. Fifth, while we focused on neuroimaging biomarkers, integrating multidimensional data such as genetics and behavioral/cognitive assessments could enhance clinical translation, as highlighted in recent frameworks (Li et al., 2023; Wang et al., 2025). These integrative approaches may help bridge the gap between underlying biological mechanisms and the heterogeneous clinical profiles seen in psychiatric disorders. Moreover, recent evidence demonstrates that neuroimaging features have potential to predict clinical prognosis and inform clinical practice (Long et al., 2025). Future studies should therefore investigate whether the shared neuroimaging features identified in RELs of SZ and BD could serve as stratification markers for early intervention in high-risk populations and predictors of disease progression trajectories. Finally, this work might not mitigate concerns with regard to small sample sizes, heterogeneous clinical populations, and methodological variability that jointly contribute to the lack of reproducibility in neuroimaging studies on psychiatry. Continued efforts will be needed to address these challenges.

In summary, the present work integrated the novel FCNM approach with large-scale human connectome data to localize brain functional and structural damage in SZ- and BD-RELs to four disorder-susceptibility networks, which showed cross-disorder inconsistencies when focusing on either imaging modality alone, but had a considerably increased spatial similarity with two modalities combined. These findings may not only support the notion that SZ and BD are distinct diagnostic categories from a neurobiological perspective, but also help to clarify the neural substrates that link the shared genetic mechanism underlying both disorders to their overlapping clinical phenotype.

## Supporting information

Yao et al. supplementary material Yao et al. supplementary material

## Data Availability

The data and analysis codes used in the preparation of this article are publicly available at https://github.com/mfmaplestory/FCNM/ .

## Supplementary material

The supplementary material for this article can be found at http://doi.org/10.1017/S0033291725101992.
