# Supplementary material for: Network localization of genetic risk for schizophrenia and bipolar disorder
Source: Psychol Med. 2025 Oct 3;55:e299. doi: 10.1017/S0033291725101992 (PMC12527508; doi:10.1017/S0033291725101992)
Supplement: Yao et al. supplementary material [file S0033291725101992sup001.docx]

**Supplementary materials**

**Supplementary methods**

**Study selection and classification**

We performed a comprehensive and systematic literature search in PubMed and Web of Science to identify relevant studies examining brain functional or structural damage in SZ- or BD-RELs, published before 1 February 2023. We used a combination of the following keywords: (schizophrenia [Title/Abstract]) OR (bipolar disorder [Title/Abstract]) AND (relative OR genetic risk OR first-degree relatives OR twins OR offspring OR parents OR high risk OR genetic risk OR liability OR family study) AND (VBM [Title/Abstract] OR fMRI [Title/Abstract] OR magnetic resonance imaging [Title/Abstract] OR MRI [Title/Abstract] OR voxel-based morphometry [Title/Abstract]). The reference lists of relevant reviews and meta-analyses were hand-searched to identify studies that were missed by the database search. Studies were included if they 1) were case-control studies; 2) included a group of unaffected first- or second-degree RELs of patients with SZ or BD, matched for age and gender with a group of HCs; 3) employed functional and/or structural magnetic resonance imaging; 4) reported stereotaxic coordinates of the differences in brain activation or volume between RELs and HCs; 5) analyzed whole-brain activation or volume. All selected studies were independently evaluated by two researchers. The initial search resulted in 5076 articles. After removing duplicates and reviewing the abstracts of these articles, 581 studies were selected for full-text reading. No direct contacts were made with the authors, as all relevant full-text articles were available. 478 studies were also excluded because: 1) imaging modalities different from task fMRI or VBM were used; 2) region of interest (ROI)-based analyses were performed; 3) participants were subjects at clinical risk for SZ or BD; 4) SZ- and BD-RELs or RELs and patients were analyzed together; 5) no stereotaxic coordinates were provided. A total of 103 studies were selected. The groups of the included studies were comparable and matched appropriately. The included studies had the following characteristics:

1. 58 fMRI studies (1-57) on 1421 SZ-RELs (mean age 30.6 years) and 1907 HCs (mean age 28.8 years);
2. 23 fMRI studies (58-80) on 592 BD-RELs (mean age 25.1 years) and 763 HCs (mean age 25.3 years);
3. 16 VBM studies (81-95) on 943 SZ-RELs (mean age 25.2 years) and 1095 HCs (mean age 26.6 years);
4. 10 VBM studies (69, 96-103) on 272 BD-RELs (mean age 30.8 years) and 349 HCs (mean age 30.6 years);

Note that the studies by Callicott et al. (2003), Guo et al. (2014), and Hajek et al. (2012) reported data from two different cohorts and were treated as independent studies. The study by Frangou et al. (2012) included both fMRI and VBM analyses that were also considered as individual studies.

**Table S1. Number and age of the participants in the selected studies**

| **Study** | **N of studies** | **SZ-RELs** | | **HCs** | | **N of studies** | **BD-RELs** | | **HCs** | |
| --- | --- | --- | --- | --- | --- | --- | --- | --- | --- | --- |
|  |  | **N** | **Age (ys)**  **mean (SD)** | **N** | **Age (ys)**  **mean (SD)** |  | **N** | **Age (ys)**  **mean (SD)** | **N** | **Age (ys)**  **mean (SD)** |
| Task fMRI | 58 | 1421 | 30.6 (6.7) | 1907 | 28.8 (6.4) | 23 | 592 | 25.1 (6.8) | 763 | 25.3 (6.2) |
| VBM | 16 | 943 | 25.2 (4.7) | 1095 | 26.6 (4.9) | 10 | 272 | 30.8 (9.2) | 349 | 30.6 (8.3) |

Abbreviations: BD, bipolar disorder; fMRI, functional magnetic resonance imaging; HCs, healthy controls; N, number; RELs, relatives; SD, standard deviation; SZ, schizophrenia; VBM, voxel-based morphometry.

**Table S2. Sample information of the fMRI studies on SZ-RELs**

| **Study** | **Kinship with SZ** | **Relatives** | | | **Healthy controls** | | | **Task** |
| --- | --- | --- | --- | --- | --- | --- | --- | --- |
|  |  | **N** | **Age (ys)**  **mean (SD)** | **M/F** | **N** | **Age (ys)**  **mean (SD)** | **M/F** |  |
| Altamura et al., 2012 | siblings | 18 | 33.3 (8.9) | 12/6 | 24 | 33.3 (8.9) | 18/6 | Visually paced variable attentional control task |
| Avsar et al., 2011 | 1^st^ degree | 5 | 36.4 (15.4) | 2/3 | 8 | 35.4 (15.4) | 4/4 | The visual delayed match-to-sample |
| Becker, Kerns, Macdonald & Carter, 2008 | 1^st^ degree | 17 | 33.3 (10.8) | 6/11 | 17 | 32.7 (7.8) | 10/7 | Stroop task |
| Bonner-Jackson, Csernansky & Barch, 2007 | siblings | 21 | 21.1 (3.5) | 10/11 | 38 | 20.9 (3.5) | 10/28 | Encoding and recognition task |
| Lo Bianco et al., 2013 | siblings | 23 | 33.9 (8.7) | 8/15 | 24 | 31.9 (3.3) | 14/10 | Emotional stimuli with negative valence |
| Brahmbhatt, Haut, Csernansky & Barch, 2006 | siblings | 18 | 20.7 (4.0) | 7/11 | 72 | 20.3 (3.5) | 34/38 | N-back working memory task |
| Callicott et al., 2003 | siblings | 23 | 34.4 (9.0) | 6/17 | 18 | 29.6 (7.0) | 11/7 | N-back working memory task |
| Callicott et al., 2003 | siblings | 25 | 36.6 (9.0) | 11/14 | 15 | 27.9 (8.0) | 6/9 | N-back working memory task |
| Choi et al., 2012 | 1^st^ degree | 17 | 20.7 (5.5) | 9/8 | 16 | 21.4 (2.3) | 9/7 | N-back working memory task |
| Collin et al., 2021 | offspring | 15 | 9.6 (2.0) | 5/10 | 19 | 9.3 (1.7) | 9/10 | Self-reference task |
| Delawalla, Csernansky & Barch, 2008 | siblings | 30 | 21.3 (3.5) | 16/14 | 92 | 20.2 (3.4) | 39/53 | Continuous performance task |
| de Leeuw, Kahn, Zandbelt, Widschwendter & Vink, 2013 | siblings | 23 | 30.1 (4.2) | 14/9 | 24 | 28.3 (5.4) | 12/12 | N-back working memory task |
| de Leeuw, Kahn & Vink, 2015 | siblings | 27 | 31.7 (1.2) | 14/13 | 29 | 30.3 (1.7) | 12/17 | Monetary incentive delay task |
| Dodell-Feder, DeLisi & Hooker, 2014 | 1^st^ degree | 19 | 27.4 (3.9) | 5/14 | 18 | 26.2 (4.0) | 4/14 | Reasoning about a story character’s thoughts task |
| Filbey, Russell, Morris, Murray & McDonald, 2008 | 1^st^ degree | 6 | 53 | 2/4 | 8 | 41 | 5/3 | Sustained & selective & dual attention task |
| Di Giorgio et al., 2013 | siblings | 48 | 36.3 (8.3) | 24/24 | 53 | 35.6 (7.5) | 25/28 | Encoding inrecognition memory |
| Grimm et al., 2014 | 1^st^ degree | 54 | 33.6 (12.4) | 23/31 | 80 | 33.5 (9.9) | 39/41 | Monetary incentive delay task |
| Gromann et al., 2014 | siblings | 50 | 33.9 (8.7) | 21/29 | 33 | 33.4 (10.2) | 19/14 | Multi-round trust game task |
| Hanssen et al., 2015 | siblings | 94 | 36.4 (10.1) | 41/53 | 57 | 32.2 (8.4) | 32/25 | Monetary incentive delay task |
| Hart et al., 2015 | 1^st^ degree | 21 | 14.4 (2.6) | 10/11 | 21 | 14.1 (2.6) | 11/10 | Emotional oddball task |
| Herold et al., 2017 | 1^st^ degree | 13 | 42.9 (10.5) | 6/6 | 12 | 37.0 (9.1) | 5/7 | Irony and control tasks |
| Jiang et al., 2015 | parents | 20 | 50.7 (5.0) | 11/9 | 20 | 51.8 (5.9) | 10/10 | N-back working memory task |
| Karch et al., 2009 | 1^st^ degree | 11 | 33.6 (8.8) | 4/7 | 11 | 33.8 (9.2) | 4/7 | N-back working memory task |
| Keshavan et al., 2002 | parents | 4 | 13.3 (2.2) | 2/2 | 4 | 12.5 (3.5) | 2/2 | Memory-guided saccade task |
| Lehet et al., 2021 | siblings | 22 | 31.1 (5.5) | 7/15 | 23 | 31.9 (8.23) | 10/13 | Saccadic stop-signal task |
| H. J. Li et al., 2012 | siblings | 12 | 31.3 (8.2) | 4/8 | 12 | 29.3 (7.2) | 6/6 | Facial emotional valence discrimination |
| Li et al., 2016 | siblings | 25 | NA | NA | 26 | NA | NA | Verbal spatial-memory task |
| Li et al., 2007 | siblings | 15 | 21.7 (6.1) | 7/8 | 15 | 23.9 (5.2) | 7/8 | Visual lexical decision task |
| Loeb et al., 2018 | siblings | 30 | 19.4 (0.8) | 13/17 | 39 | 20.0 (0.7) | 18/21 | N-back working memory task |
| Lopez-Garcia et al., 2016 | 1^st^ degree | 16 | 57.1 (10.3) | 9/7 | 20 | 32.7 (11.2) | 8/12 | Dot probe expectancy task |
| MacDonald, Becker & Carter, 2006 | 1^st^ degree | 21 | 33.2 (10.9) | 7/14 | 20 | 33.4 (8.4) | 10/10 | Stimulus-response incompatibility task |
| McAllindon, Wilman, Purdon & Tibbo, 2010 | 1^st^ degree | 11 | 34.7 | 11/0 | 14 | 36.4 | NA | Visual 2-choice reaction time experiment |
| Nielsen et al., 2023 | siblings | 34 | 39.5 (11.3) | 16/18 | 93 | 41.3 (10.3) | 42/41 | Monetary incentive delay task |
| Nook et al., 2018 | 1^st^ degree | 21 | 27.3 (3.9) | 7/14 | 19 | 26.0 (3.9) | 5/14 | Expressed emotion task |
| Oertel et al., 2019 | 1^st^ degree | 23 | 43.6 (14.3) | NA | 27 | 34.2 (11.4) | NA | Face–name association paradigm |
| Park et al., 2016 | 1^st^ degree | 20 | 23.9 (5.6) | 7/13 | 17 | 23.1 (3.9) | 8/9 | Facial emotion recognition task |
| Pirnia et al., 2015 | 1^st^ degree | 14 | 39.6 (11.8) | 5/9 | 30 | 29.3 (9.0) | 24/6 | Declarative memory task |
| Pulkkinen et al., 2015 | offspring | 51 | 22.4 (0.8) | 20/31 | 52 | 22.3 (0.7) | 20/32 | Facial emotion recognition task |
| Raemaekers, Ramsey, Vink, van den Heuvel & Kahn, 2006 | siblings | 16 | 33.9 (11.3) | 8/8 | 16 | 33.4 (13.6) | 8/8 | Prosaccades and antisaccades |
| Rajarethinam, Venkatesh, Peethala, Phan & Keshavan, 2011 | offspring | 15 | 15.1 (3.4) | 7/8 | 17 | 14.5 (3.5) | 9/8 | Listen to 30 sec blocks of a story alternated |
| Rasetti et al., 2014 | siblings | 65 | 36.0 (1.2) | 27/38 | 181 | 34.9 (0.7) | 86/95 | Declarative memory task |
| Sambataro et al., 2013 | siblings | 65 | 36.6 (10.4) | 24/41 | 235 | 31.8 (9.6) | 113/122 | Flanker task |
| Seidman et al., 2007 | 1^st^ degree | 12 | 34.8 (8.2) | 5/7 | 13 | 36.9 (8.1) | 6/7 | N-back working memory task |
| Seidman et al., 2006 | 1^st^ degree | 21 | 19.9 (4.0) | 12/9 | 24 | 18.1 (3.3) | 10/14 | N-back working memory task |
| Sepede et al., 2010 | siblings | 11 | 34.4 (8.8) | 5/6 | 11 | 32.0 (5.1) | 5/6 | Continuous performance test |
| Spilka & Goghari, 2017 | 1^st^ degree | 27 | 41.2 (15.5) | 10/17 | 27 | 40.7 (11.1) | 13/14 | Facial emotion recognition task |
| Spilka, Arnold & Goghari, 2015 | 1^st^ degree | 27 | 41.2 (15.5) | 10/17 | 27 | 40.7 (11.1) | 13/14 | Passive viewing facial emotion perception task |
| Stäblein et al., 2019 | 1^st^ degree | 22 | 42.7 (14.9) | 8/14 | 25 | 34.9 (10.5) | 12/13 | Visual masked change detection task |
| Stolz et al., 2012 | 1^st^ degree | 16 | 23.0 (5.1) | 6/10 | 28 | 26.9 (6.9) | 9/19 | Visual episodic memory encoding and retrieval task |
| Thermenos et al., 2004 | 1^st^ degree | 12 | 35.5 (6.0) | 4/8 | 12 | 32.2 (7.7) | 6/6 | Auditory working memory task |
| Thermenos et al., 2007 | 1^st^ degree | 21 | 19.9 (4.0) | 12/9 | 26 | 18.0 (3.2) | 10/16 | Miller Selfridge context memory test |
| Thermenos et al., 2013 | 1^st^ degree | 43 | 25.2 (3.1) | 12/31 | 32 | 24.6 (2.8) | 13/19 | Lexical decision task |
| van Buuren, Vink & Kahn, 2012 | siblings | 25 | 27.9 (4.6) | 9/16 | 25 | 27.5 (8.1) | 9/16 | Self-referential task |
| van der Meer et al., 2014 | siblings | 20 | 32.6 (8.6) | 11/9 | 20 | 35.5 (11.7) | 14/6 | Emotion regulation task |
| van Gool et al., 2022 | 1^st^ degree | 15 | 9.6 (2.1) | 6/9 | 18 | 9.3 (1.7) | 8/10 | N-back working memory task |
| Venkatasubramanian, Puthumana, Jayakumar & Gangadhar, 2010 | siblings | 17 | 25.2 (4.2) | 14/3 | 16 | 24.4 (3.7) | 14/2 | Facial emotion recognition task |
| Whitfield-Gabrieli et al., 2009 | 1^st^ degree | 13 | 22.0 (2.9) | 10/3 | 13 | 20.5 (3.3) | 8/5 | N-back working memory task |
| Whyte et al., 2006 | 1^st^ or 2^nd^ degree | 41 | 26.6 (3.3) | 18/23 | 21 | 26.8 (2.7) | 13/8 | Verbal classification and recognition task |

Abbreviations: F, female; fMRI, functional magnetic resonance imaging; M, male; N, number; NA, not available; SD, standard deviation; SZ, schizophrenia.

**Table S3. Sample information of the fMRI studies on BD-RELs**

| **Study** | **Kinship with BD** | **Relatives** | | | **Healthy controls** | | | **Task** |
| --- | --- | --- | --- | --- | --- | --- | --- | --- |
|  |  | **N** | **Age (ys)**  **mean (SD)** | **M/F** | **N** | **Age (ys)**  **mean (SD)** | **M/F** |  |
| Allin et al., 2010 | 1^st^ degree | 19 | 40.5 (13.9) | 8/11 | 19 | 39.9 (11.0) | 9/10 | Verbal fluency task |
| Alonso-Lana et al., 2016 | siblings | 20 | 43.8 (11.1) | 6/14 | 40 | 42.4 (10.7) | 11/29 | N-back working memory task |
| Chan et al., 2016 | 1^st^ degree | 43 | 23.8 (2.5) | 24/19 | 54 | 23.0 (2.4) | 21/33 | Explicit facial expression recognition task |
| K. Chang et al., 2017 | offspring | 50 | 13.5 (2.9) | 29/21 | 29 | 13.6 (2.8) | 15/14 | Facial expressions task |
| Frangou, 2011 | 1^st^ degree | 48 | 36.5 (13.8) | 25/23 | 71 | 39.8 (15.3) | 35/36 | Cognitive control task |
| Kanske, Schönfelder, Forneck & Wessa, 2015 | 1^st^ degree | 17 | 36.7 (16.3) | 8/9 | 17 | 35.9 (15.6) | 8/9 | Facial emotion recognition task |
| Kim et al., 2012 | 1^st^ degree | 13 | 13.9 (2.0) | 6/7 | 21 | 13.7 (2.0) | 13/8 | Change task |
| Linke et al., 2012 | 1^st^ degree | 22 | 28.0 (11.0) | 11/11 | 22 | 28.0 (10.0) | 11/11 | Probabilistic reversal learning task |
| Manelis et al., 2015 | offspring | 29 | 13.8 (2.4) | 17/12 | 23 | 13.7 (1.8) | 11/12 | Dynamic faces task |
| Manelis et al., 2016 | offspring | 29 | 13.8 (2.5) | 15/14 | 28 | 13.9 (2.4) | 16/12 | Number guessing reward task |
| Nimarko, Gorelik, Carta, Gorelik & Singh, 2022 | offspring | 40 | 12.5 (2.8) | 14/26 | 45 | 13.2 (2.7) | 18/27 | Monetary incentive delay task |
| Nimarko et al., 2021 | offspring | 29 | 13.0 (2.7) | 11/18 | 28 | 14.6 (2.5) | 12/16 | Implicit emotion perception task |
| Olsavsky et al., 2012 | 1^st^ degree | 13 | 14.0 (2.4) | 7/6 | 56 | 14.0 (2.6) | 26/30 | Facial emotion recognition task |
| Pagliaccio et al., 2017 | 1^st^ degree | 29 | 14.9 (3.5) | 15/14 | 24 | 19.1 (3.8) | 9/15 | Global-local selective attention task |
| Pompei et al., 2011 | 1^st^ degree | 25 | 35.0 (13.7) | 13/12 | 48 | 36.3 (12.8) | 25/23 | Stroop colour word task |
| Roberts et al., 2013 | 1^st^ degree | 47 | 24.6 (3.8) | 22/25 | 49 | 23.2 (3.4) | 17/32 | Facial emotion go/no-go task |
| Sepede et al., 2012 | 1^st^ degree | 22 | 31.5 (7.3) | 7/15 | 24 | 32.5 (6.2) | 8/16 | Continuous performance test |
| Sugihara et al., 2017 | MZ twins | 14 | 40.6 (14.5) | 3/11 | 48 | 35.6 (11.4) | 10/38 | N-back working memory task |
| Surguladze et al., 2010 | 1^st^ degree | 20 | 43.0 (13.8) | 12/8 | 20 | 41.9 (11.6) | 10/10 | Facial emotion recognition task |
| Tseng et al., 2015 | 1^st^ degree | 13 | 13.7 (2.3) | 8/5 | 27 | 14.4 (2.8) | 15/12 | Facial emotion recognition task |
| Thermenos et al., 2011 | 1^st^ degree | 10 | 18.4 (4.2) | 5/5 | 10 | 17.1 (1.4) | 5/5 | N-back working memory task |
| Thermenos et al., 2010 | 1^st^ degree | 18 | 36.3 (2.6) | 8/10 | 19 | 39.2 (2.7) | 9/10 | N-back working memory task |
| Wiggins et al., 2017 | 1^st^ degree | 22 | 15.7 (3.6) | 13/9 | 41 | 17.3 (4.2) | 21/20 | Face emotion labeling task |

Abbreviations: BD, bipolar disorder; F, female; fMRI, functional magnetic resonance imaging; M, male; MZ twins, monozygotic twins; N, number; SD, standard deviation.

**Table S4. Sample information of the VBM studies on SZ-RELs**

| **Study** | **Kinship with SZ** | **Relatives** | | | **Healthy controls** | | |
| --- | --- | --- | --- | --- | --- | --- | --- |
|  |  | **N** | **Age (ys)**  **mean (SD)** | **M/F** | **N** | **Age (ys)**  **mean (SD)** | **M/F** |
| M. Chang et al., 2016 | offspring | 31 | 18.4 (3.8) | 21/10 | 71 | 20.6 (3.5) | 27/44 |
| Guo et al., 2014 | siblings | 20 | 23.3 (3.4) | 14/6 | 43 | 23.7 (2.8) | 25/18 |
| Guo et al., 2014 | siblings | 25 | 23.0 (4.5) | 17/8 | 43 | 23.7 (2.8) | 25/18 |
| Guo et al., 2015 | siblings | 46 | 23.0 (4.0) | 29/17 | 46 | 23.3 (2.3) | 23/23 |
| Honea et al., 2008 | siblings | 213 | 36.5 (9.8) | 89/114 | 212 | 33.3 (9.9) | 103/109 |
| Hu et al., 2013 | 1^st^ degree | 45 | 22.6 (3.9) | 29/16 | 59 | 23.2 (2.6) | 38/21 |
| Ivleva et al., 2017 | 1^st^ degree | 200 | NA | NA | 251 | 36.9 (12.1) | 88/163 |
| Job et al., 2003 | 1^st^ or 2^nd^ degree | 146 | 21.2 (2.9) | 74/72 | 36 | 21.2 (2.4) | 17/19 |
| X. Li et al., 2012 | 1^st^ degree | 21 | 21.1 (5.5) | 7/14 | 48 | 22.0 (5.1) | 24/24 |
| B. Lin et al., 2022 | 1^st^ degree | 18 | 23.9 (4.8) | 5/13 | 54 | 25.9 (4.6) | 34/20 |
| Lui et al., 2009 | parents | 10 | 41.4 (3.7) | 3/7 | 10 | 43.2 (6.3) | 5/5 |
| Marcelis et al., 2003 | 1^st^ degree | 32 | 35.5 (10.0) | 14/18 | 27 | 35.5 (9.8) | 12/15 |
| Oertel-Knöchel et al., 2012 | 1^st^ degree | 29 | 15.1 (4.1) | 14/15 | 37 | 16.1 (2.6) | 17/20 |
| Sugranyes et al., 2015 | offspring | 38 | 11.0 (3.3) | 25/13 | 83 | 11.8 (3.2) | 39/44 |
| Tian et al., 2011 | parents | 55 | 50.3 (5.1) | 27/28 | 29 | 51.8 (5.6) | 14/15 |
| Wagshal et al., 2015 | siblings | 14 | 12.1 (2.4) | 6/8 | 46 | 12.9 (2.6) | 21/25 |

Abbreviations: F, female; M, male; N, number; N, number; NA, not available; SD, standard deviation; SZ, schizophrenia; VBM, voxel-based morphometry.

**Table S5. Sample information of the VBM studies on BD-RELs**

| **Study** | **Kinship with BD** | **Relatives** | | | **Healthy controls** | | |
| --- | --- | --- | --- | --- | --- | --- | --- |
|  |  | **N** | **Age (ys)**  **mean (SD)** | **M/F** | **N** | **Age (ys)**  **mean (SD)** | **M/F** |
| Frangou, 2011 | 1^st^ degree | 48 | 36.5 (13.8) | 25/23 | 71 | 39.8 (15.3) | 36/35 |
| Eker et al., 2014 | 1^st^ degree | 30 | 34.7 (8.4) | 10/20 | 28 | 34.9 (9.4) | 11/17 |
| Hajek et al., 2013 | offspring | 30 | 19.5 (3.1) | 10/20 | 31 | 20.6 (3.3) | 11/20 |
| Hajek et al., 2013 | offspring | 20 | 20.2 (4.2) | 9/11 | 18 | 23.0 (3.5) | 7/11 |
| Hanford, Hall, Minuzzi & Sassi, 2016 | offspring | 13 | 12.5 (3.0) | 8/5 | 20 | 13.3 (2.5) | 11/9 |
| Kempton et al., 2009 | 1^st^ degree | 50 | 33.8 (12.7) | 24/26 | 52 | 35.2 (13.0) | 27/25 |
| K. Lin et al., 2015 | offspring | 26 | 17.7 (5.4) | 11/15 | 33 | 15.9 (4.4) | 15/18 |
| Matsubara et al., 2016 | 1^st^ degree | 10 | 54.8 (20.1) | 5/5 | 27 | 48.3 (13.0) | 10/17 |
| Matsuo et al., 2012 | 1^st^ degree | 20 | 46.2 (10.7) | 16/8 | 40 | 41.6 (9.1) | 16/24 |
| Sarıçiçek et al., 2015 | 1^st^ degree | 25 | 32.1 (11.0) | 12/13 | 29 | 33.6 (9.3) | 13/16 |

Abbreviations: BD, bipolar disorder; F, female; M, male; N, number; SD, standard deviation; VBM, voxel-based morphometry.

**Table S6. Demographic information of AMUD and SALD**

| **Dataset** | **Sample size** | **Age (years)** | **Gender (F/M)** | **FD (mm)** |
| --- | --- | --- | --- | --- |
| AMUD | 656 | 26.57 (8.57) | 396/260 | 0.12 (0.06) |
| SALD | 329 | 37.81 (13.79) | 207/122 | 0.15 (0.08) |

Age and FD are expressed as mean (standard deviation). Abbreviations: AMUD, Anhui Medical University Dataset; F, female; FD, frame-wise displacement; M, male; SALD, Southwest University Adult Lifespan Dataset.

**Table S7. Resting-state fMRI parameters of AMUD and SALD**

| **Parameter** | **AMUD** | **SALD** |
| --- | --- | --- |
| Scanner | 3.0T General Electric Discovery MR750w | 3.0T Siemens Trio |
| Sequence | GRE-SS-EPI | GRE-EPI |
| TR (ms) | 2000 | 2000 |
| TE (ms) | 30 | 30 |
| FA (°) | 90 | 90 |
| FOV (mm2) | 220 × 220 | 220 × 220 |
| Matrix size | 64 × 64 | 64 × 64 |
| Slice thickness (mm) | 3 | 3 |
| Slice gap (mm) | 1 | 1 |
| Slices | 35 | 32 |
| Time points | 185 | 242 |

Abbreviations: AMUD, Anhui Medical University Dataset; EPI, echo planar imaging; FA, flip angle; fMRI, functional magnetic resonance imaging; FOV, field of view; GRE, gradient echo; SALD, Southwest University Adult Lifespan Dataset; SS, single shot; TE, echo time; TR, repetition time.

Studies meeting all inclusion criteria = 103

Met exclusion criteria = 2624

Publications potentially meeting inclusion criteria = 581

Records after duplicates removed = 3205

Records identified through database screens = 5076

Full-text publications assessed for eligibility = 581

Excluded following studies = 478

1. Imaging modalities different from task fMRI or VBM: 177
2. ROI-based studies: 94
3. Subjects at clinical risk for SZ or BD: 92
4. SZ- and BD-RELs or RELs and patients analyzed together: 27
5. No stereotaxic coordinates were provided: 88

**Figure S1.** The study selection process. Abbreviations: BD, bipolar disorder; fMRI, functional magnetic resonance imaging; RELs, relatives; ROI, region of interest; SZ, schizophrenia; VBM, voxel-based morphometry.


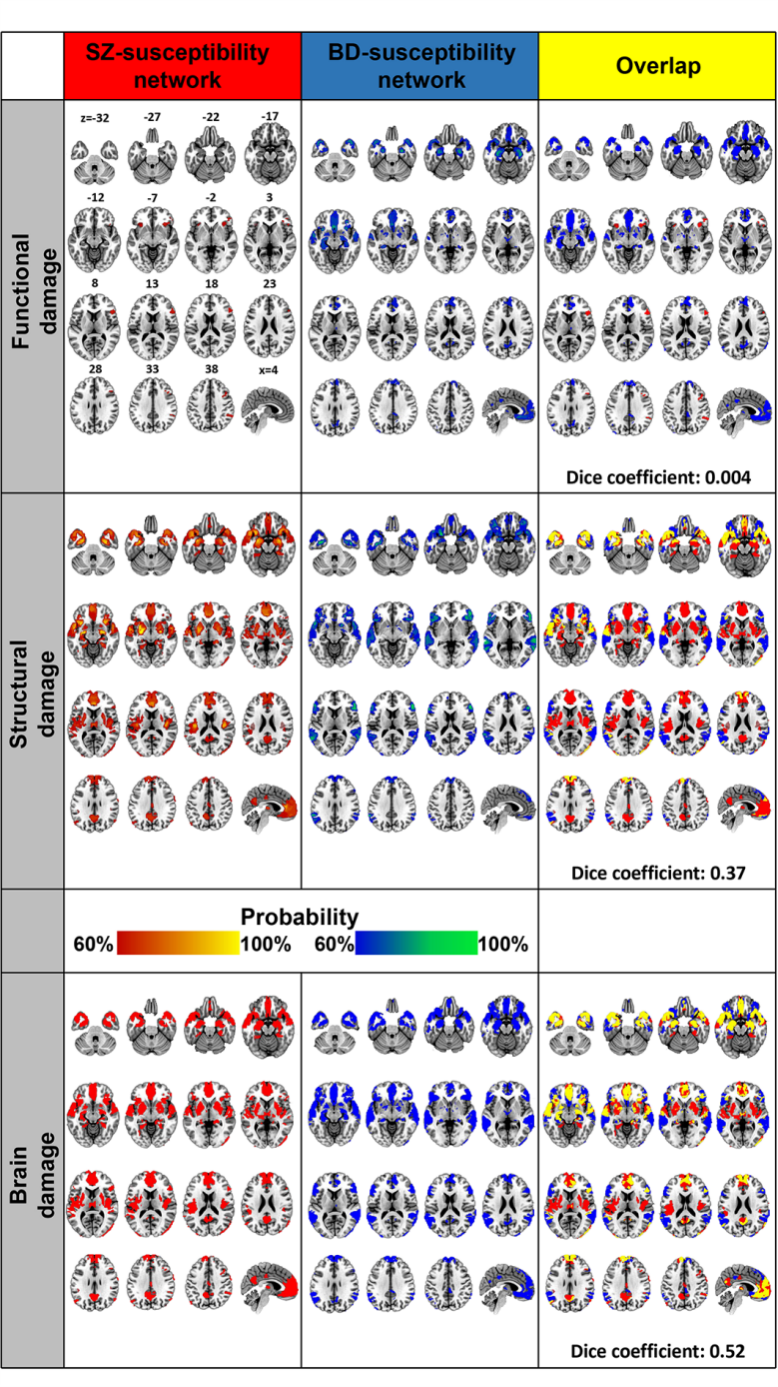


**Figure S2.** Schizophrenia and bipolar disorder susceptibility networks derived from the SALD dataset. Left panel: SZ-susceptibility functional, structural, and combined brain damage networks. Middle panel: BD-susceptibility functional, structural, and combined brain damage networks. Right panel: spatial overlap between SZ- and BD-susceptibility networks. Abbreviations: BD, bipolar disorder; SALD, Southwest University Adult Lifespan Dataset; SZ, schizophrenia.


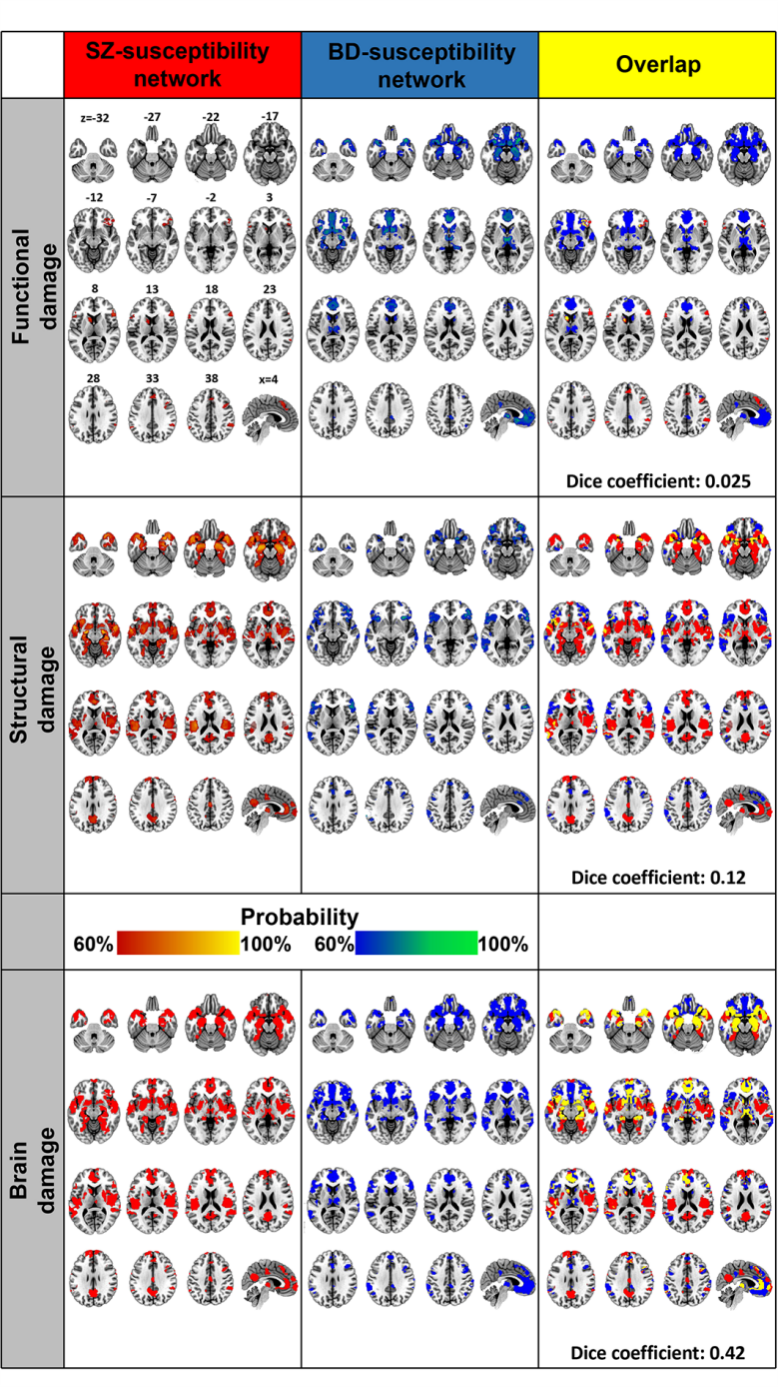


**Figure S3.** Schizophrenia and bipolar disorder susceptibility networks based on 1-mm radius sphere. Left panel: SZ-susceptibility functional, structural, and combined brain damage networks. Middle panel: BD-susceptibility functional, structural, and combined brain damage networks. Right panel: spatial overlap between SZ- and BD-susceptibility networks. Abbreviations: BD, bipolar disorder; SZ, schizophrenia.


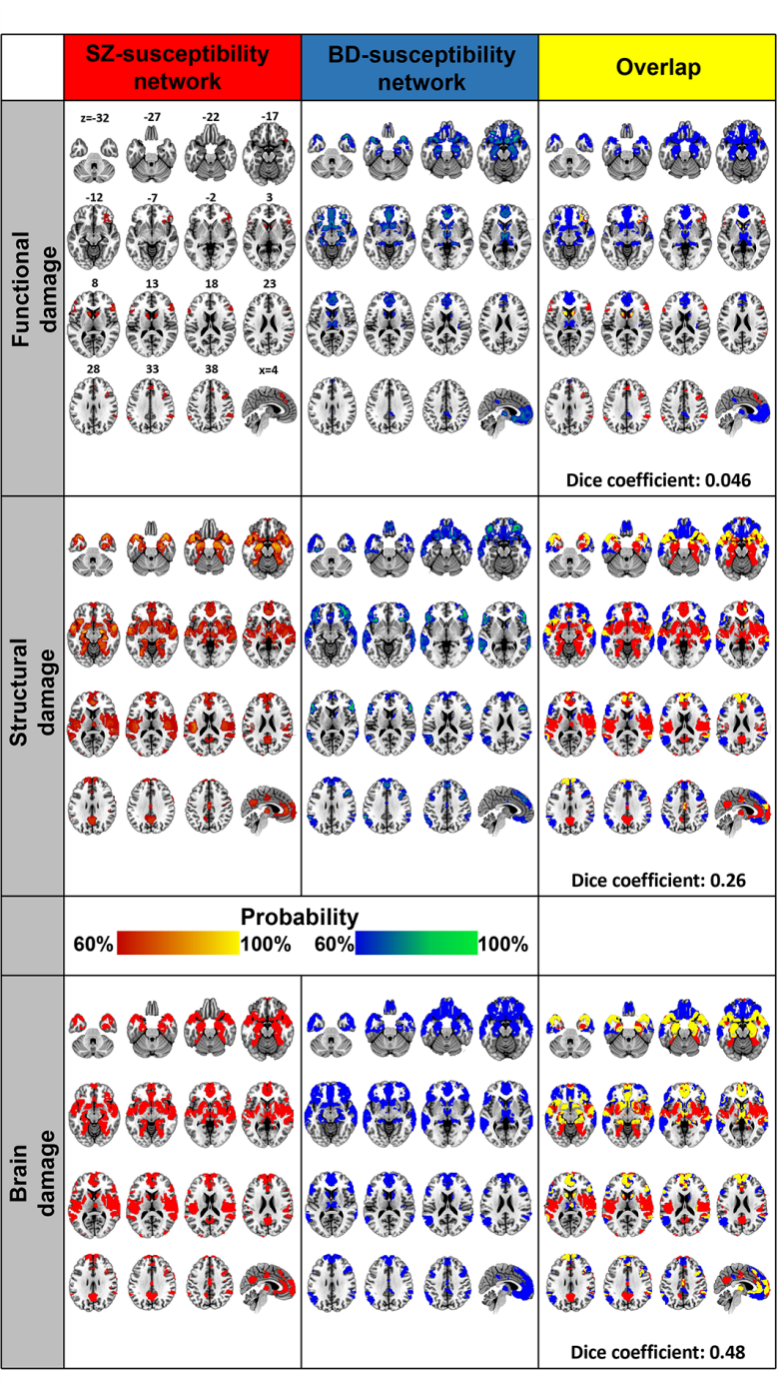


**Figure S4.** Schizophrenia and bipolar disorder susceptibility networks based on 7-mm radius sphere. Left panel: SZ-susceptibility functional, structural, and combined brain damage networks. Middle panel: BD-susceptibility functional, structural, and combined brain damage networks. Right panel: spatial overlap between SZ- and BD-susceptibility networks. Abbreviations: BD, bipolar disorder; SZ, schizophrenia.


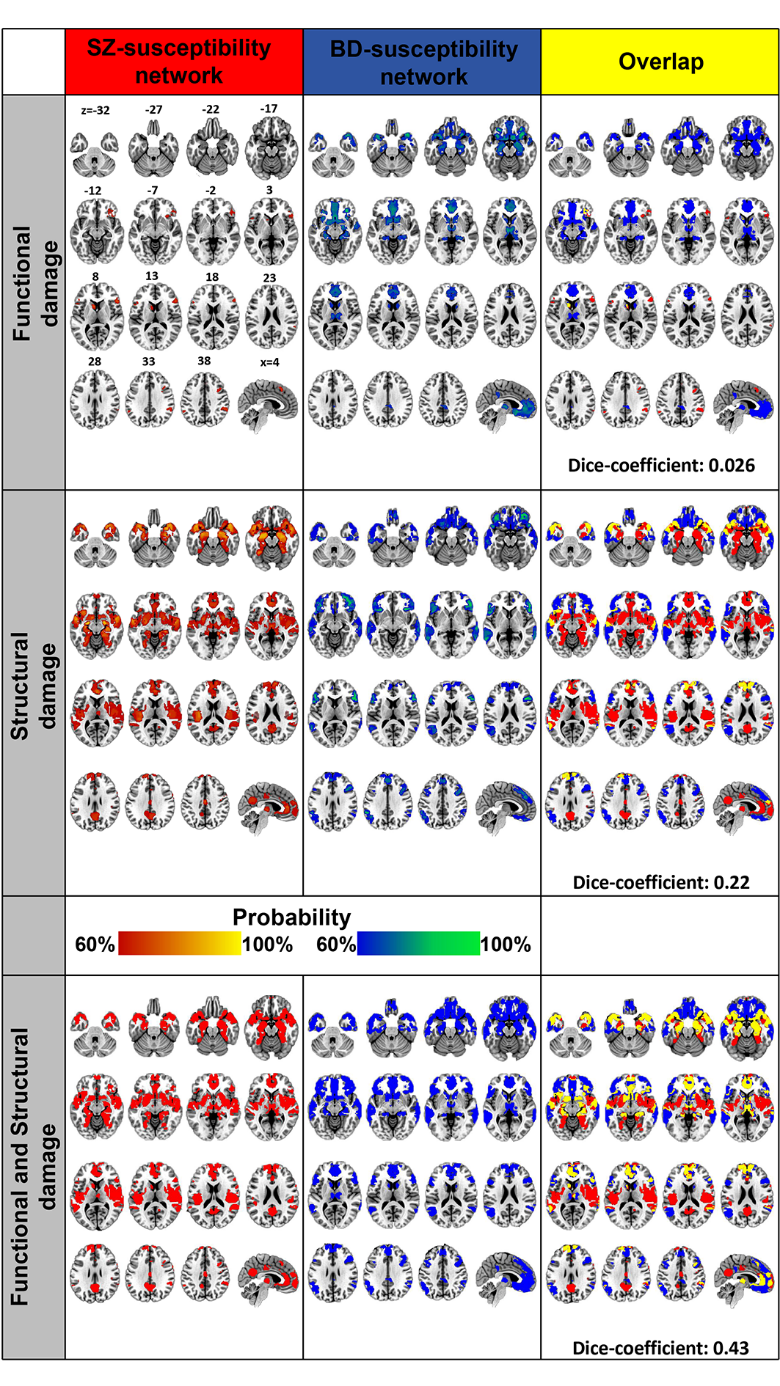


**Figure S5.** Schizophrenia and bipolar disorder susceptibility networks derived from the young adults within an age range of 18-30 years. Left panel: SZ-susceptibility functional, structural, and combined brain damage networks. Middle panel: BD-susceptibility functional, structural, and combined brain damage networks. Right panel: spatial overlap between SZ- and BD-susceptibility networks. Abbreviations: BD, bipolar disorder; SZ, schizophrenia.**References**

Allin M. P., Marshall N., Schulze K., Walshe M., Hall M. H., Picchioni M., ... McDonald C. (2010). A functional MRI study of verbal fluency in adults with bipolar disorder and their unaffected relatives. *Psychol Med*, 40(12), 2025-2035. doi: 10.1017/s0033291710000127

Alonso-Lana S., Valentí M., Romaguera A., Sarri C., Sarró S., Rodríguez-Martínez A., ... Pomarol-Clotet E. (2016). Brain functional changes in first-degree relatives of patients with bipolar disorder: evidence for default mode network dysfunction. *Psychol Med*, 46(12), 2513-2521. doi: 10.1017/s0033291716001148

Altamura M., Fazio L., De Salvia M., Petito A., Blasi G., Taurisano P., ... Bertolino A. (2012). Abnormal functional motor lateralization in healthy siblings of patients with schizophrenia. *Psychiatry Res*, 203(1), 54-60. doi: 10.1016/j.pscychresns.2012.02.008

Avsar K. B., Stoeckel L. E., Bolding M. S., White D. M., Tagamets M. A., Holcomb H. H., & Lahti A. C. (2011). Aberrant visual circuitry associated with normal spatial match-to-sample accuracy in schizophrenia. *Psychiatry Res*, 193(3), 138-143. doi: 10.1016/j.pscychresns.2011.03.008

Becker T. M., Kerns J. G., Macdonald A. W., 3rd, & Carter C. S. (2008). Prefrontal dysfunction in first-degree relatives of schizophrenia patients during a Stroop task. *Neuropsychopharmacology*, 33(11), 2619-2625. doi: 10.1038/sj.npp.1301673

Bonner-Jackson A., Csernansky J. G., & Barch D. M. (2007). Levels-of-processing effects in first-degree relatives of individuals with schizophrenia. *Biol Psychiatry*, 61(10), 1141-1147. doi: 10.1016/j.biopsych.2006.07.006

Brahmbhatt S. B., Haut K., Csernansky J. G., & Barch D. M. (2006). Neural correlates of verbal and nonverbal working memory deficits in individuals with schizophrenia and their high-risk siblings. *Schizophr Res*, 87(1-3), 191-204. doi: 10.1016/j.schres.2006.05.019

Callicott J. H., Egan M. F., Mattay V. S., Bertolino A., Bone A. D., Verchinksi B., & Weinberger D. R. (2003). Abnormal fMRI response of the dorsolateral prefrontal cortex in cognitively intact siblings of patients with schizophrenia. *Am J Psychiatry*, 160(4), 709-719. doi: 10.1176/appi.ajp.160.4.709

Chan S. W., Sussmann J. E., Romaniuk L., Stewart T., Lawrie S. M., Hall J., ... Whalley H. C. (2016). Deactivation in anterior cingulate cortex during facial processing in young individuals with high familial risk and early development of depression: fMRI findings from the Scottish Bipolar Family Study. *J Child Psychol Psychiatry*, 57(11), 1277-1286. doi: 10.1111/jcpp.12591

Chang K., Garrett A., Kelley R., Howe M., Sanders E. M., Acquaye T., ... Reiss A. (2017). Anomalous prefrontal-limbic activation and connectivity in youth at high-risk for bipolar disorder. *J Affect Disord*, 222, 7-13. doi: 10.1016/j.jad.2017.05.051

Chang M., Womer F. Y., Bai C., Zhou Q., Wei S., Jiang X., ... Wang F. (2016). Voxel-Based Morphometry in Individuals at Genetic High Risk for Schizophrenia and Patients with Schizophrenia during Their First Episode of Psychosis. *PLoS One*, 11(10), e0163749. doi: 10.1371/journal.pone.0163749

Choi J. S., Park J. Y., Jung M. H., Jang J. H., Kang D. H., Jung W. H., ... Kwon J. S. (2012). Phase-specific brain change of spatial working memory processing in genetic and ultra-high risk groups of schizophrenia. *Schizophr Bull*, 38(6), 1189-1199. doi: 10.1093/schbul/sbr038

Collin G., Bauer C. C. C., Anteraper S. A., Gabrieli J. D. E., Molokotos E., Mesholam-Gately R., ... Whitfield-Gabrieli S. (2021). Hyperactivation of Posterior Default Mode Network During Self-Referential Processing in Children at Familial High-Risk for Psychosis. *Front Psychiatry*, 12, 613142. doi: 10.3389/fpsyt.2021.613142

de Leeuw M., Kahn R. S., & Vink M. (2015). Fronto-striatal dysfunction during reward processing in unaffected siblings of schizophrenia patients. *Schizophr Bull*, 41(1), 94-103. doi: 10.1093/schbul/sbu153

de Leeuw M., Kahn R. S., Zandbelt B. B., Widschwendter C. G., & Vink M. (2013). Working memory and default mode network abnormalities in unaffected siblings of schizophrenia patients. *Schizophr Res*, 150(2-3), 555-562. doi: 10.1016/j.schres.2013.08.016

Delawalla Z., Csernansky J. G., & Barch D. M. (2008). Prefrontal cortex function in nonpsychotic siblings of individuals with schizophrenia. *Biol Psychiatry*, 63(5), 490-497. doi: 10.1016/j.biopsych.2007.05.007

Di Giorgio A., Gelao B., Caforio G., Romano R., Andriola I., D'Ambrosio E., ... Bertolino A. (2013). Evidence that hippocampal-parahippocampal dysfunction is related to genetic risk for schizophrenia. *Psychol Med*, 43(8), 1661-1671. doi: 10.1017/s0033291712002413

Dodell-Feder D., DeLisi L. E., & Hooker C. I. (2014). Neural disruption to theory of mind predicts daily social functioning in individuals at familial high-risk for schizophrenia. *Soc Cogn Affect Neurosci*, 9(12), 1914-1925. doi: 10.1093/scan/nst186

Eker C., Simsek F., Yılmazer E. E., Kitis O., Cinar C., Eker O. D., ... Gonul A. S. (2014). Brain regions associated with risk and resistance for bipolar I disorder: a voxel-based MRI study of patients with bipolar disorder and their healthy siblings. *Bipolar Disord*, 16(3), 249-261. doi: 10.1111/bdi.12181

Filbey F. M., Russell T., Morris R. G., Murray R. M., & McDonald C. (2008). Functional magnetic resonance imaging (fMRI) of attention processes in presumed obligate carriers of schizophrenia: preliminary findings. *Ann Gen Psychiatry*, 7, 18. doi: 10.1186/1744-859x-7-18

Frangou S. (2011). Brain structural and functional correlates of resilience to bipolar disorder. *Front Hum Neurosci*, 5, 184. doi: 10.3389/fnhum.2011.00184

Grimm O., Heinz A., Walter H., Kirsch P., Erk S., Haddad L., ... Meyer-Lindenberg A. (2014). Striatal response to reward anticipation: evidence for a systems-level intermediate phenotype for schizophrenia. *JAMA Psychiatry*, 71(5), 531-539. doi: 10.1001/jamapsychiatry.2014.9

Gromann P. M., Shergill S. S., de Haan L., Meewis D. G., Fett A. K., Korver-Nieberg N., & Krabbendam L. (2014). Reduced brain reward response during cooperation in first-degree relatives of patients with psychosis: an fMRI study. *Psychol Med*, 44(16), 3445-3454. doi: 10.1017/s0033291714000737

Guo W., Hu M., Fan X., Liu F., Wu R., Chen J., ... Zhao J. (2014). Decreased gray matter volume in the left middle temporal gyrus as a candidate biomarker for schizophrenia: a study of drug naive, first-episode schizophrenia patients and unaffected siblings. *Schizophr Res*, 159(1), 43-50. doi: 10.1016/j.schres.2014.07.051

Guo W., Song Y., Liu F., Zhang Z., Zhang J., Yu M., ... Zhao J. (2015). Dissociation of functional and anatomical brain abnormalities in unaffected siblings of schizophrenia patients. *Clin Neurophysiol*, 126(5), 927-932. doi: 10.1016/j.clinph.2014.08.016

Hajek T., Cullis J., Novak T., Kopecek M., Blagdon R., Propper L., ... Alda M. (2013). Brain structural signature of familial predisposition for bipolar disorder: replicable evidence for involvement of the right inferior frontal gyrus. *Biol Psychiatry*, 73(2), 144-152. doi: 10.1016/j.biopsych.2012.06.015

Hanford L. C., Hall G. B., Minuzzi L., & Sassi R. B. (2016). Gray matter volumes in symptomatic and asymptomatic offspring of parents diagnosed with bipolar disorder. *Eur Child Adolesc Psychiatry*, 25(9), 959-967. doi: 10.1007/s00787-015-0809-y

Hanssen E., van der Velde J., Gromann P. M., Shergill S. S., de Haan L., Bruggeman R., ... van Atteveldt N. (2015). Neural correlates of reward processing in healthy siblings of patients with schizophrenia. *Front Hum Neurosci*, 9, 504. doi: 10.3389/fnhum.2015.00504

Hart S. J., Shaffer J. J., Bizzell J., Weber M., McMahon M. A., Gu H., ... Belger A. (2015). Measurement of Fronto-limbic Activity Using an Emotional Oddball Task in Children with Familial High Risk for Schizophrenia. *J Vis Exp*(106). doi: 10.3791/51484

Herold R., Varga E., Hajnal A., Hamvas E., Berecz H., Tóth B., & Tényi T. (2017). Altered Neural Activity during Irony Comprehension in Unaffected First-Degree Relatives of Schizophrenia Patients-An fMRI Study. *Front Psychol*, 8, 2309. doi: 10.3389/fpsyg.2017.02309

Honea R. A., Meyer-Lindenberg A., Hobbs K. B., Pezawas L., Mattay V. S., Egan M. F., ... Callicott J. H. (2008). Is gray matter volume an intermediate phenotype for schizophrenia? A voxel-based morphometry study of patients with schizophrenia and their healthy siblings. *Biol Psychiatry*, 63(5), 465-474. doi: 10.1016/j.biopsych.2007.05.027

Hu M., Li J., Eyler L., Guo X., Wei Q., Tang J., ... Zhao J. (2013). Decreased left middle temporal gyrus volume in antipsychotic drug-naive, first-episode schizophrenia patients and their healthy unaffected siblings. *Schizophr Res*, 144(1-3), 37-42. doi: 10.1016/j.schres.2012.12.018

Ivleva E. I., Clementz B. A., Dutcher A. M., Arnold S. J. M., Jeon-Slaughter H., Aslan S., ... Tamminga C. A. (2017). Brain Structure Biomarkers in the Psychosis Biotypes: Findings From the Bipolar-Schizophrenia Network for Intermediate Phenotypes. *Biol Psychiatry*, 82(1), 26-39. doi: 10.1016/j.biopsych.2016.08.030

Jiang S., Yan H., Chen Q., Tian L., Lu T., Tan H. Y., ... Zhang D. (2015). Cerebral Inefficient Activation in Schizophrenia Patients and Their Unaffected Parents during the N-Back Working Memory Task: A Family fMRI Study. *PLoS One*, 10(8), e0135468. doi: 10.1371/journal.pone.0135468

Job D. E., Whalley H. C., McConnell S., Glabus M., Johnstone E. C., & Lawrie S. M. (2003). Voxel-based morphometry of grey matter densities in subjects at high risk of schizophrenia. *Schizophr Res*, 64(1), 1-13. doi: 10.1016/s0920-9964(03)00158-0

Kanske P., Schönfelder S., Forneck J., & Wessa M. (2015). Impaired regulation of emotion: neural correlates of reappraisal and distraction in bipolar disorder and unaffected relatives. *Transl Psychiatry*, 5(1), e497. doi: 10.1038/tp.2014.137

Karch S., Leicht G., Giegling I., Lutz J., Kunz J., Buselmeier M., ... Mulert C. (2009). Inefficient neural activity in patients with schizophrenia and nonpsychotic relatives of schizophrenic patients: evidence from a working memory task. *J Psychiatr Res*, 43(15), 1185-1194. doi: 10.1016/j.jpsychires.2009.04.004

Kempton M. J., Haldane M., Jogia J., Grasby P. M., Collier D., & Frangou S. (2009). Dissociable brain structural changes associated with predisposition, resilience, and disease expression in bipolar disorder. *J Neurosci*, 29(35), 10863-10868. doi: 10.1523/jneurosci.2204-09.2009

Keshavan M. S., Diwadkar V. A., Spencer S. M., Harenski K. A., Luna B., & Sweeney J. A. (2002). A preliminary functional magnetic resonance imaging study in offspring of schizophrenic parents. *Prog Neuropsychopharmacol Biol Psychiatry*, 26(6), 1143-1149. doi: 10.1016/s0278-5846(02)00249-x

Kim P., Jenkins S. E., Connolly M. E., Deveney C. M., Fromm S. J., Brotman M. A., ... Leibenluft E. (2012). Neural correlates of cognitive flexibility in children at risk for bipolar disorder. *J Psychiatr Res*, 46(1), 22-30. doi: 10.1016/j.jpsychires.2011.09.015

Lehet M., Tso I. F., Park S., Neggers S. F. W., Thompson I. A., Kahn R. S., & Thakkar K. N. (2021). Altered Effective Connectivity within an Oculomotor Control Network in Unaffected Relatives of Individuals with Schizophrenia. *Brain Sci*, 11(9). doi: 10.3390/brainsci11091228

Li H. J., Chan R. C., Gong Q. Y., Liu Y., Liu S. M., Shum D., & Ma Z. L. (2012). Facial emotion processing in patients with schizophrenia and their non-psychotic siblings: a functional magnetic resonance imaging study. *Schizophr Res*, 134(2-3), 143-150. doi: 10.1016/j.schres.2011.10.019

Li X., Alapati V., Jackson C., Xia S., Bertisch H. C., Branch C. A., & Delisi L. E. (2012). Structural abnormalities in language circuits in genetic high-risk subjects and schizophrenia patients. *Psychiatry Res*, 201(3), 182-189. doi: 10.1016/j.pscychresns.2011.07.017

Li X., Branch C. A., Bertisch H. C., Brown K., Szulc K. U., Ardekani B. A., & DeLisi L. E. (2007). An fMRI study of language processing in people at high genetic risk for schizophrenia. *Schizophr Res*, 91(1-3), 62-72. doi: 10.1016/j.schres.2006.12.016

Li X., Thermenos H. W., Wu Z., Momura Y., Wu K., Keshavan M., ... DeLisi L. E. (2016). Abnormal interactions of verbal- and spatial-memory networks in young people at familial high-risk for schizophrenia. *Schizophr Res*, 176(2-3), 100-105. doi: 10.1016/j.schres.2016.07.022

Lin B., Li X. B., Ruan S., Wu Y. X., Zhang C. Y., Wang C. Y., & Wang L. B. (2022). Convergent and divergent gray matter volume abnormalities in unaffected first-degree relatives and ultra-high risk individuals of schizophrenia. *Schizophrenia (Heidelb)*, 8(1), 55. doi: 10.1038/s41537-022-00261-9

Lin K., Xu G., Wong N. M., Wu H., Li T., Lu W., ... Lee T. M. (2015). A Multi-Dimensional and Integrative Approach to Examining the High-Risk and Ultra-High-Risk Stages of Bipolar Disorder. *EBioMedicine*, 2(8), 919-928. doi: 10.1016/j.ebiom.2015.06.027

Linke J., King A. V., Rietschel M., Strohmaier J., Hennerici M., Gass A., ... Wessa M. (2012). Increased medial orbitofrontal and amygdala activation: evidence for a systems-level endophenotype of bipolar I disorder. *Am J Psychiatry*, 169(3), 316-325. doi: 10.1176/appi.ajp.2011.11050711

Lo Bianco L., Blasi G., Taurisano P., Di Giorgio A., Ferrante F., Ursini G., ... Bertolino A. (2013). Interaction between catechol-O-methyltransferase (COMT) Val158Met genotype and genetic vulnerability to schizophrenia during explicit processing of aversive facial stimuli. *Psychol Med*, 43(2), 279-292. doi: 10.1017/s0033291712001134

Loeb F. F., Zhou X., Craddock K. E. S., Shora L., Broadnax D. D., Gochman P., ... Liu S. (2018). Reduced Functional Brain Activation and Connectivity During a Working Memory Task in Childhood-Onset Schizophrenia. *J Am Acad Child Adolesc Psychiatry*, 57(3), 166-174. doi: 10.1016/j.jaac.2017.12.009

Lopez-Garcia P., Cristobal-Huerta A., Young Espinoza L., Molero P., Ortuño Sanchez-Pedreño F., & Hernández-Tamames J. A. (2016). The influence of the COMT genotype in the underlying functional brain activity of context processing in schizophrenia and in relatives. *Prog Neuropsychopharmacol Biol Psychiatry*, 71, 176-182. doi: 10.1016/j.pnpbp.2016.07.005

Lui S., Deng W., Huang X., Jiang L., Ouyang L., Borgwardt S. J., ... Gong Q. (2009). Neuroanatomical differences between familial and sporadic schizophrenia and their parents: an optimized voxel-based morphometry study. *Psychiatry Res*, 171(2), 71-81. doi: 10.1016/j.pscychresns.2008.02.004

MacDonald A. W., 3rd, Becker T. M., & Carter C. S. (2006). Functional magnetic resonance imaging study of cognitive control in the healthy relatives of schizophrenia patients. *Biol Psychiatry*, 60(11), 1241-1249. doi: 10.1016/j.biopsych.2006.04.041

Manelis A., Ladouceur C. D., Graur S., Monk K., Bonar L. K., Hickey M. B., ... Phillips M. L. (2016). Altered functioning of reward circuitry in youth offspring of parents with bipolar disorder. *Psychol Med*, 46(1), 197-208. doi: 10.1017/s003329171500166x

Manelis A., Ladouceur C. D., Graur S., Monk K., Bonar L. K., Hickey M. B., ... Phillips M. L. (2015). Altered amygdala-prefrontal response to facial emotion in offspring of parents with bipolar disorder. *Brain*, 138(Pt 9), 2777-2790. doi: 10.1093/brain/awv176

Marcelis M., Suckling J., Woodruff P., Hofman P., Bullmore E., & van Os J. (2003). Searching for a structural endophenotype in psychosis using computational morphometry. *Psychiatry Res*, 122(3), 153-167. doi: 10.1016/s0925-4927(02)00125-7

Matsubara T., Matsuo K., Harada K., Nakano M., Nakashima M., Watanuki T., ... Watanabe Y. (2016). Distinct and Shared Endophenotypes of Neural Substrates in Bipolar and Major Depressive Disorders. *PLoS One*, 11(12), e0168493. doi: 10.1371/journal.pone.0168493

Matsuo K., Kopecek M., Nicoletti M. A., Hatch J. P., Watanabe Y., Nery F. G., ... Soares J. C. (2012). New structural brain imaging endophenotype in bipolar disorder. *Mol Psychiatry*, 17(4), 412-420. doi: 10.1038/mp.2011.3

McAllindon D. P., Wilman A. H., Purdon S. E., & Tibbo P. G. (2010). Functional magnetic resonance imaging of choice reaction time in chronic schizophrenia and first-degree relatives. *Schizophr Res*, 120(1-3), 232-233. doi: 10.1016/j.schres.2010.01.015

Nielsen M., Rostrup E., Hilker R., Legind C., Anhøj S., Robbins T. W., ... Glenthøj B. (2023). Reward Processing as an Indicator of Vulnerability or Compensatory Resilience in Psychoses? Results From a Twin Study. *Biol Psychiatry Glob Open Sci*, 3(1), 47-55. doi: 10.1016/j.bpsgos.2022.01.002

Nimarko A. F., Fischer A. S., Hagan K. E., Gorelik A. J., Lu Y., Young C. J., & Singh M. K. (2021). Neural Correlates of Positive Emotion Processing That Distinguish Healthy Youths at Familial Risk for Bipolar Versus Major Depressive Disorder. *J Am Acad Child Adolesc Psychiatry*, 60(7), 887-901. doi: 10.1016/j.jaac.2020.07.890

Nimarko A. F., Gorelik A. J., Carta K. E., Gorelik M. G., & Singh M. K. (2022). Neural correlates of reward processing distinguish healthy youth at familial risk for bipolar disorder from youth at familial risk for major depressive disorder. *Transl Psychiatry*, 12(1), 31. doi: 10.1038/s41398-022-01800-9

Nook E. C., Dodell-Feder D., Germine L. T., Hooley J. M., DeLisi L. E., & Hooker C. I. (2018). Weak dorsolateral prefrontal response to social criticism predicts worsened mood and symptoms following social conflict in people at familial risk for schizophrenia. *Neuroimage Clin*, 18, 40-50. doi: 10.1016/j.nicl.2018.01.004

Oertel-Knöchel V., Knöchel C., Matura S., Rotarska-Jagiela A., Magerkurth J., Prvulovic D., ... Linden D. E. (2012). Cortical-basal ganglia imbalance in schizophrenia patients and unaffected first-degree relatives. *Schizophr Res*, 138(2-3), 120-127. doi: 10.1016/j.schres.2012.02.029

Oertel V., Kraft D., Alves G., Knöchel C., Ghinea D., Storchak H., ... Stäblein M. (2019). Associative Memory Impairments Are Associated with Functional Alterations Within the Memory Network in Schizophrenia Patients and Their Unaffected First-Degree Relatives: An fMRI Study. *Front Psychiatry*, 10, 33. doi: 10.3389/fpsyt.2019.00033

Olsavsky A. K., Brotman M. A., Rutenberg J. G., Muhrer E. J., Deveney C. M., Fromm S. J., ... Leibenluft E. (2012). Amygdala hyperactivation during face emotion processing in unaffected youth at risk for bipolar disorder. *J Am Acad Child Adolesc Psychiatry*, 51(3), 294-303. doi: 10.1016/j.jaac.2011.12.008

Pagliaccio D., Wiggins J. L., Adleman N. E., Harkins E., Curhan A., Towbin K. E., ... Leibenluft E. (2017). Behavioral and Neural Sustained Attention Deficits in Bipolar Disorder and Familial Risk of Bipolar Disorder. *Biol Psychiatry*, 82(9), 669-678. doi: 10.1016/j.biopsych.2016.09.006

Park H. Y., Yun J. Y., Shin N. Y., Kim S. Y., Jung W. H., Shin Y. S., ... Kwon J. S. (2016). Decreased neural response for facial emotion processing in subjects with high genetic load for schizophrenia. *Prog Neuropsychopharmacol Biol Psychiatry*, 71, 90-96. doi: 10.1016/j.pnpbp.2016.06.014

Pirnia T., Woods R. P., Hamilton L. S., Lyden H., Joshi S. H., Asarnow R. F., ... Narr K. L. (2015). Hippocampal dysfunction during declarative memory encoding in schizophrenia and effects of genetic liability. *Schizophr Res*, 161(2-3), 357-366. doi: 10.1016/j.schres.2014.11.030

Pompei F., Jogia J., Tatarelli R., Girardi P., Rubia K., Kumari V., & Frangou S. (2011). Familial and disease specific abnormalities in the neural correlates of the Stroop Task in Bipolar Disorder. *Neuroimage*, 56(3), 1677-1684. doi: 10.1016/j.neuroimage.2011.02.052

Pulkkinen J., Nikkinen J., Kiviniemi V., Mäki P., Miettunen J., Koivukangas J., ... Veijola J. (2015). Functional mapping of dynamic happy and fearful facial expressions in young adults with familial risk for psychosis - Oulu Brain and Mind Study. *Schizophr Res*, 164(1-3), 242-249. doi: 10.1016/j.schres.2015.01.039

Raemaekers M., Ramsey N. F., Vink M., van den Heuvel M. P., & Kahn R. S. (2006). Brain activation during antisaccades in unaffected relatives of schizophrenic patients. *Biol Psychiatry*, 59(6), 530-535. doi: 10.1016/j.biopsych.2005.07.030

Rajarethinam R., Venkatesh B. K., Peethala R., Phan K. L., & Keshavan M. (2011). Reduced activation of superior temporal gyrus during auditory comprehension in young offspring of patients with schizophrenia. *Schizophr Res*, 130(1-3), 101-105. doi: 10.1016/j.schres.2011.05.025

Rasetti R., Mattay V. S., White M. G., Sambataro F., Podell J. E., Zoltick B., ... Weinberger D. R. (2014). Altered hippocampal-parahippocampal function during stimulus encoding: a potential indicator of genetic liability for schizophrenia. *JAMA Psychiatry*, 71(3), 236-247. doi: 10.1001/jamapsychiatry.2013.3911

Roberts G., Green M. J., Breakspear M., McCormack C., Frankland A., Wright A., ... Mitchell P. B. (2013). Reduced inferior frontal gyrus activation during response inhibition to emotional stimuli in youth at high risk of bipolar disorder. *Biol Psychiatry*, 74(1), 55-61. doi: 10.1016/j.biopsych.2012.11.004

Sambataro F., Mattay V. S., Thurin K., Safrin M., Rasetti R., Blasi G., ... Weinberger D. R. (2013). Altered cerebral response during cognitive control: a potential indicator of genetic liability for schizophrenia. *Neuropsychopharmacology*, 38(5), 846-853. doi: 10.1038/npp.2012.250

Sarıçiçek A., Yalın N., Hıdıroğlu C., Çavuşoğlu B., Taş C., Ceylan D., ... Özerdem A. (2015). Neuroanatomical correlates of genetic risk for bipolar disorder: A voxel-based morphometry study in bipolar type I patients and healthy first degree relatives. *J Affect Disord*, 186, 110-118. doi: 10.1016/j.jad.2015.06.055

Seidman L. J., Thermenos H. W., Koch J. K., Ward M., Breiter H., Goldstein J. M., ... Tsuang M. T. (2007). Auditory verbal working memory load and thalamic activation in nonpsychotic relatives of persons with schizophrenia: an fMRI replication. *Neuropsychology*, 21(5), 599-610. doi: 10.1037/0894-4105.21.5.599

Seidman L. J., Thermenos H. W., Poldrack R. A., Peace N. K., Koch J. K., Faraone S. V., & Tsuang M. T. (2006). Altered brain activation in dorsolateral prefrontal cortex in adolescents and young adults at genetic risk for schizophrenia: an fMRI study of working memory. *Schizophr Res*, 85(1-3), 58-72. doi: 10.1016/j.schres.2006.03.019

Sepede G., De Berardis D., Campanella D., Perrucci M. G., Ferretti A., Serroni N., ... Gambi F. (2012). Impaired sustained attention in euthymic bipolar disorder patients and non-affected relatives: an fMRI study. *Bipolar Disord*, 14(7), 764-779. doi: 10.1111/bdi.12007

Sepede G., Ferretti A., Perrucci M. G., Gambi F., Di Donato F., Nuccetelli F., ... Romani G. L. (2010). Altered brain response without behavioral attention deficits in healthy siblings of schizophrenic patients: an event-related fMRI study. *Neuroimage*, 49(1), 1080-1090. doi: 10.1016/j.neuroimage.2009.07.053

Spilka M. J., Arnold A. E., & Goghari V. M. (2015). Functional activation abnormalities during facial emotion perception in schizophrenia patients and nonpsychotic relatives. *Schizophr Res*, 168(1-2), 330-337. doi: 10.1016/j.schres.2015.07.012

Spilka M. J., & Goghari V. M. (2017). Similar patterns of brain activation abnormalities during emotional and non-emotional judgments of faces in a schizophrenia family study. *Neuropsychologia*, 96, 164-174. doi: 10.1016/j.neuropsychologia.2017.01.014

Stäblein M., Storchak H., Ghinea D., Kraft D., Knöchel C., Prvulovic D., ... Oertel-Knöchel V. (2019). Visual working memory encoding in schizophrenia and first-degree relatives: neurofunctional abnormalities and impaired consolidation. *Psychol Med*, 49(1), 75-83. doi: 10.1017/s003329171800051x

Stolz E., Pancholi K. M., Goradia D. D., Paul S., Keshavan M. S., Nimgaonkar V. L., & Prasad K. M. (2012). Brain activation patterns during visual episodic memory processing among first-degree relatives of schizophrenia subjects. *Neuroimage*, 63(3), 1154-1161. doi: 10.1016/j.neuroimage.2012.08.030

Sugihara G., Kane F., Picchioni M. M., Chaddock C. A., Kravariti E., Kalidindi S., ... McGuire P. (2017). Effects of risk for bipolar disorder on brain function: A twin and family study. *Eur Neuropsychopharmacol*, 27(5), 494-503. doi: 10.1016/j.euroneuro.2017.03.001

Sugranyes G., de la Serna E., Romero S., Sanchez-Gistau V., Calvo A., Moreno D., ... Castro-Fornieles J. (2015). Gray Matter Volume Decrease Distinguishes Schizophrenia from Bipolar Offspring During Childhood and Adolescence. *J Am Acad Child Adolesc Psychiatry*, 54(8), 677-684.e672. doi: 10.1016/j.jaac.2015.05.003

Surguladze S. A., Marshall N., Schulze K., Hall M. H., Walshe M., Bramon E., ... McDonald C. (2010). Exaggerated neural response to emotional faces in patients with bipolar disorder and their first-degree relatives. *Neuroimage*, 53(1), 58-64. doi: 10.1016/j.neuroimage.2010.05.069

Thermenos H. W., Goldstein J. M., Milanovic S. M., Whitfield-Gabrieli S., Makris N., Laviolette P., ... Seidman L. J. (2010). An fMRI study of working memory in persons with bipolar disorder or at genetic risk for bipolar disorder. *Am J Med Genet B Neuropsychiatr Genet*, 153b(1), 120-131. doi: 10.1002/ajmg.b.30964

Thermenos H. W., Makris N., Whitfield-Gabrieli S., Brown A. B., Giuliano A. J., Lee E. H., ... Seidman L. J. (2011). A functional MRI study of working memory in adolescents and young adults at genetic risk for bipolar disorder: preliminary findings. *Bipolar Disord*, 13(3), 272-286. doi: 10.1111/j.1399-5618.2011.00920.x

Thermenos H. W., Seidman L. J., Breiter H., Goldstein J. M., Goodman J. M., Poldrack R., ... Tsuang M. T. (2004). Functional magnetic resonance imaging during auditory verbal working memory in nonpsychotic relatives of persons with schizophrenia: a pilot study. *Biol Psychiatry*, 55(5), 490-500. doi: 10.1016/j.biopsych.2003.11.014

Thermenos H. W., Seidman L. J., Poldrack R. A., Peace N. K., Koch J. K., Faraone S. V., & Tsuang M. T. (2007). Elaborative verbal encoding and altered anterior parahippocampal activation in adolescents and young adults at genetic risk for schizophrenia using FMRI. *Biol Psychiatry*, 61(4), 564-574. doi: 10.1016/j.biopsych.2006.04.044

Thermenos H. W., Whitfield-Gabrieli S., Seidman L. J., Kuperberg G., Juelich R. J., Divatia S., ... DeLisi L. E. (2013). Altered language network activity in young people at familial high-risk for schizophrenia. *Schizophr Res*, 151(1-3), 229-237. doi: 10.1016/j.schres.2013.09.023

Tian L., Meng C., Yan H., Zhao Q., Liu Q., Yan J., ... Zhang D. (2011). Convergent evidence from multimodal imaging reveals amygdala abnormalities in schizophrenic patients and their first-degree relatives. *PLoS One*, 6(12), e28794. doi: 10.1371/journal.pone.0028794

Tseng W. L., Bones B. L., Kayser R. R., Olsavsky A. K., Fromm S. J., Pine D. S., ... Brotman M. A. (2015). An fMRI study of emotional face encoding in youth at risk for bipolar disorder. *Eur Psychiatry*, 30(1), 94-98. doi: 10.1016/j.eurpsy.2014.05.004

van Buuren M., Vink M., & Kahn R. S. (2012). Default-mode network dysfunction and self-referential processing in healthy siblings of schizophrenia patients. *Schizophr Res*, 142(1-3), 237-243. doi: 10.1016/j.schres.2012.09.017

van der Meer L., Swart M., van der Velde J., Pijnenborg G., Wiersma D., Bruggeman R., & Aleman A. (2014). Neural correlates of emotion regulation in patients with schizophrenia and non-affected siblings. *PLoS One*, 9(6), e99667. doi: 10.1371/journal.pone.0099667

van Gool K. C. A., Collin G., Bauer C. C. C., Molokotos E., Mesholam-Gately R. I., Thermenos H. W., ... Keshavan M. S. (2022). Altered working memory-related brain activity in children at familial high risk for psychosis: A preliminary study. *Schizophr Res*, 240, 186-192. doi: 10.1016/j.schres.2021.12.030

Venkatasubramanian G., Puthumana D. T., Jayakumar P. N., & Gangadhar B. N. (2010). A functional Magnetic Resonance Imaging study of neurohemodynamic abnormalities during emotion processing in subjects at high risk for schizophrenia. *Indian J Psychiatry*, 52(4), 308-315. doi: 10.4103/0019-5545.74304

Wagshal D., Knowlton B. J., Cohen J. R., Bookheimer S. Y., Bilder R. M., Fernandez V. G., & Asarnow R. F. (2015). Cognitive correlates of gray matter abnormalities in adolescent siblings of patients with childhood-onset schizophrenia. *Schizophr Res*, 161(2-3), 345-350. doi: 10.1016/j.schres.2014.12.006

Whitfield-Gabrieli S., Thermenos H. W., Milanovic S., Tsuang M. T., Faraone S. V., McCarley R. W., ... Seidman L. J. (2009). Hyperactivity and hyperconnectivity of the default network in schizophrenia and in first-degree relatives of persons with schizophrenia. *Proc Natl Acad Sci U S A*, 106(4), 1279-1284. doi: 10.1073/pnas.0809141106

Whyte M. C., Whalley H. C., Simonotto E., Flett S., Shillcock R., Marshall I., ... Lawrie S. M. (2006). Event-related fMRI of word classification and successful word recognition in subjects at genetically enhanced risk of schizophrenia. *Psychol Med*, 36(10), 1427-1439. doi: 10.1017/s0033291706008178

Wiggins J. L., Brotman M. A., Adleman N. E., Kim P., Wambach C. G., Reynolds R. C., ... Leibenluft E. (2017). Neural Markers in Pediatric Bipolar Disorder and Familial Risk for Bipolar Disorder. *J Am Acad Child Adolesc Psychiatry*, 56(1), 67-78. doi: 10.1016/j.jaac.2016.10.009
